# A Sustainable Approach to the Stereoselective Synthesis of Diazaheptacyclic Cage Systems Based on a Multicomponent Strategy in an Ionic Liquid

**DOI:** 10.3390/molecules21020165

**Published:** 2016-01-29

**Authors:** Raju Suresh Kumar, Abdulrahman I. Almansour, Natarajan Arumugam, Mohammad Altaf, José Carlos Menéndez, Raju Ranjith Kumar, Hasnah Osman

**Affiliations:** 1 Department of Chemistry, College of Science, King Saud University P. O. Box 2455, Riyadh 11451, Saudi Arabia; almansor@ksu.edu.sa (A.I.A.); anatarajan@ksu.edu.sa (N.A.); 2 Central Laboratory, College of Science, King Saud University P. O. Box 2455, Riyadh 11451, Saudi Arabia; altafamu@gmail.com; 3 Departamento de Química Orgánica y Farmacéutica, Facultad de Farmacia, Universidad Complutense, Madrid 28040, Spain; josecm@farm.ucm.es; 4 Department of Organic Chemistry, School of Chemistry, Madurai Kamaraj University, Madurai 625 021, Tamil Nadu, India; raju.ranjithkumar@gmail.com; 5 School of Chemical Sciences, Universiti Sains Malaysia, Minden 11800, Penang, Malaysia; ohasnah@usm.my

**Keywords:** diazaheptacyclic cage compounds, multicomponent reactions, ionic liquid, microwave-assisted synthesis

## Abstract

The microwave-assisted three-component reactions of 3,5-bis(*E*)-arylmethylidene]tetrahydro-4(1*H*)-pyridinones, acenaphthenequinone and cyclic α-amino acids in an ionic liquid, 1-butyl-3-methylimidazolium bromide, occurred through a domino sequence affording structurally intriguing diazaheptacyclic cage-like compounds in excellent yields.

## 1. Introduction

Achieving molecular complexity and diversity from common starting materials with a minimum number of synthetic steps and short reaction time is a major challenge for synthetic chemists [1,2,3,4,5]. Multi-component reactions (MCR) have proven to be one of the most effective and attractive methods to achieve this goal [6,7,8]. These reactions allow several bond-forming or bond-breaking transformations to occur in a single step, thereby obviating the time-consuming and costly processes of isolation or purification of various intermediates formed in each steps, and also the tedious operations of protection or deprotection of functional groups. Consequently, these reactions are environmentally benign and often proceed with excellent stereoselectivities [9]. Therefore, the design of new selective MCRs for the synthesis of diverse heterocycles of biological significance is a continuing challenge at the forefront of synthetic organic chemistry.

On the other hand, the choice of an appropriate reaction medium is crucial for a successful synthesis. Recently, more emphasis has been focused on the use of eco-friendly solvents. In this regard, ionic liquids are widely recognized as “green” solvents as an alternative to the volatile organic solvents and are suitable for executing diverse organic reactions [10,11]. The development of multicomponent reactions in ionic liquids, although relatively unexplored [12], is of great interest.

Furthermore, microwave-assisted reactions have been reported to proceed in dramatically shortened reaction times as compared to reactions under conventional heating. Under these conditions, the reactions are usually cleaner, affording enhanced product yields and avoiding the formation of unnecessary side products. Microwave-assisted synthesis has significant advantages in several chemical transformations [13,14], including cycloadditions [15].

**Scheme 1:**
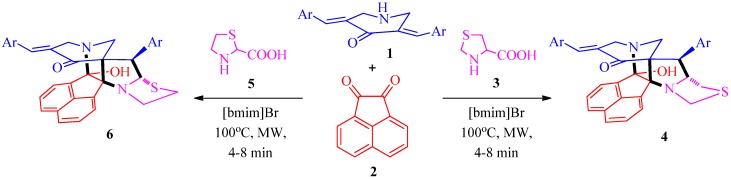
Synthesis of diazaheptacycles **4** and **6**.

The synthesis of cage-like compounds has received considerable attention in view of their biological activities and applications as artificial receptors [16]. Gambogic acid, a naturally occurring cage-like compound has been identified as a potent antitumor agent [17] and has recently finished phase IIa clinical trials [18]. The biological evaluation of Gambogic acid derivatives indicated that the peripheral moieties were suitable sites for diverse modification while the α,β-unsaturated moiety in the caged ring was essential for antitumor activity [19]. A recent study from our laboratory revealed that several polycyclic cage compounds embedded with an α,β-unsaturated moiety displayed promising AChE inhibitory activity [20,21]. Several reports pertaining to the synthesis of polycyclic caged structures are available in the literature. However, these methods have mostly relied on multi-step sequences and therefore the development of greener, step-economic routes is imperative. Herein we report the stereoselective synthesis of structurally diverse heptacyclic cage-like frameworks from the three-component domino reactions of 3,5-bis(*E*)-arylmethylidene]tetrahydro-4(1*H*)-pyridinones **1**, acenaphthenequinone **2** and cyclic α-amino acids **3** or **5** in ionic liquid under microwave conditions (Scheme 1) with the aim of studying their pharmacological profiles in the near future. Furthermore, the present work also stems from our ongoing investigation aimed at synthesizing novel heterocycles employing green chemical protocols [20,21,22,23,24,25,26,27,28,29,30,31].

## 2. Results and Discussion

Initially, the precursor 3,5-bis[(*E*)-arylmethylidene]tetrahydro-4(1*H*)-pyridinones **1** was synthesized following a literature method [32]. Then, the optimized reaction condition established in our earlier report for the synthesis of analogous cage-like compounds [23] was employed in the present investigation for the synthesis of two series of novel diazaheptacycles **4** and **6** (Scheme 1 and Table 1). In a typical reaction, an equimolar mixture of the starting materials **1**, **2** and **3** or **5** in 100 mg of ionic liquid [bmim]Br was subjected to microwave irradiation at 100 °C for 4–8 min (Scheme 1 and Table 1). After completion of the reaction, the products **4** and **6** were isolated by extraction and crystallization. After extraction of the product, the ionic liquid [bmim]Br was dried under vacuum, and its recyclability was investigated by successive syntheses of compounds **4** or **6**, which revealed that its efficacy was not significantly diminished after up to three subsequent runs. Furthermore, these reactions gain importance from the viewpoint of green chemistry as the crude reaction products were clean enough to be purified just by crystallization, thereby eliminating the need for chromatography, the main source of waste from synthetic activities.

**Table 1:** Reaction time, yield and melting point of diazaheptacycles **4a**–**n** and **6a**–**m**.

Entry	Ar	Comp.	Reaction Time (min)	Yield (%) ^a^	m.p. (°C)	Comp.	Reaction Time (min)	Yield (%) ^a^	m.p. (°C)
1	C_6_H_5_	**4a**	4	92	140–142	**6a**	4	93	135–137
2	2-CH_3_C_6_H_4_	**4b**	6	90	127–129	**6b**	6	90	179–181
3	2-OCH_3_C_6_H_4_	**4c**	8	85	166–168	**6c**	8	84	175–177
4	2-BrC_6_H_4_	**4d**	4	91	184–186	**6d**	4	92	172–174
5	2-ClC_6_H_4_	**4e**	4	90	146–148	**6e**	6	87	134–136
6	2-FC_6_H_4_	**4f**	6	88	150–152	**6f**	6	90	162–164
7	3-NO_2_C_6_H_4_	**4g**	6	91	176–178	**6g**	4	89	180–182
8	2,4-Cl_2_C_6_H_3_	**4h**	4	90	144–146	**6h**	6	91	146–147
9	4-CH_3_C_6_H_4_	**4i**	4	93	141–143	**6i**	6	92	190–192
10	4-OCH_3_C_6_H_4_	**4j**	6	87	137–139	**6j**	6	86	144–146
11	4-BrC_6_H_4_	**4k**	4	92	171–173	**6k**	4	90	156–158
12	4-ClC_6_H_4_	**4l**	4	95	154–156	**6l**	4	92	164–166
13	4-FC_6_H_4_	**4m**	6	93	132–134	**6m**	6	91	136–138
14	1-Naphthyl	**4n**	6	89	158–160	-	-	-	-

^a^ Isolated yield.

**Scheme 2:**
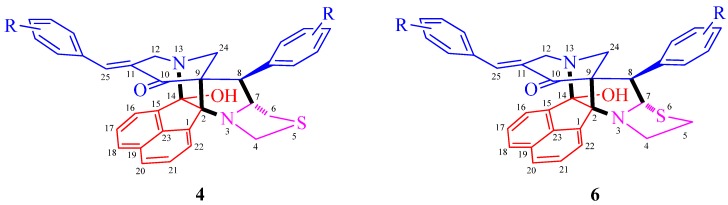
Arbitrary atom numbering of **4** and **6**.

**Scheme 3:**
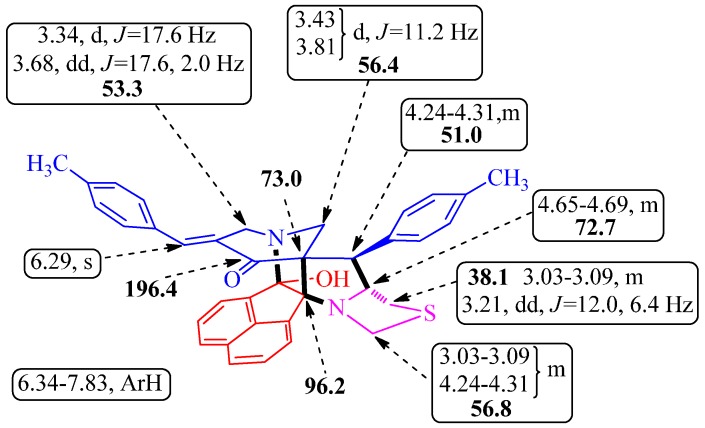
Selected ^1^H- and **^13^C**-NMR chemical shifts of **4i**.

The arbitrary atom numbering of heptacyclic cage compounds **4** and **6** are shown in Scheme 2. The structures of cage compounds **4** and **6** were elucidated using Infrared (IR) and Nuclear Magnetic Resonance (NMR) spectroscopic studies (vide Supplementary Materials) as discussed for **4i**. In the IR spectrum, the absorptions at 3416, 1723, 1682 and 1594 cm^−1^ were attributed to the O-H, C=H (arylmethylidene), C=O and C=H (aromatic ring) stretching frequency, respectively. In the ^1^H-NMR spectrum of **4i**, the singlet at 6.29 ppm was readily assigned to the arylmethylidene proton (H-25) on the basis of its multiplicity. Furthermore, H-25 showed HMBCs with the carbon signal at 53.3 ppm assignable to C-12 besides showing correlation with C-10 and C-11, the *ipso* and *ortho* carbons of the *p*-methylphenyl ring. From the C,H-COSY correlation of C-12, the doublet at 3.34 ppm (*J* 17.6 Hz) and the doublet of doublets at 3.68 ppm (*J* 17.6, 2.0 Hz) was assigned to H-12a and H-12b, respectively. The other doublets at 3.43 ppm and 3.81 ppm (*J* 11.2 Hz) were due to H-24a and H-24b. The multiplet in the range 4.24–4.31 ppm was due to H-8 and the C,H-COSY assigned the carbon at 51.0 ppm to C-8. The multiplet in the range 4.65–4.69 ppm was assigned to H-7 as it showed H,H-COSY with H-8. The multiplet in the range 3.03–3.09 ppm accounting for two protons was assigned to H-4a and H-6a. The doublet of doublets at 3.21 ppm (*J* 12.0, 6.4 Hz) was assigned to H-6b, whereas H-4b appeared as multiplet in the range 4.24–4.31 ppm. The two CH_3_ protons appeared as singlets at 2.23 and 2.33 ppm and the aromatic protons appeared as multiplet in the range 6.34–7.83 ppm. The carbon signals at 73.0 and 96.2 ppm was assigned to the spiro-carbons C-9 and C-2, respectively (Scheme 3). Similarly, the structure of the other heptacyclic cage-like compounds **6** was also assigned using NMR spectroscopy and X-ray crystallographic studies.

**Figure 1:**
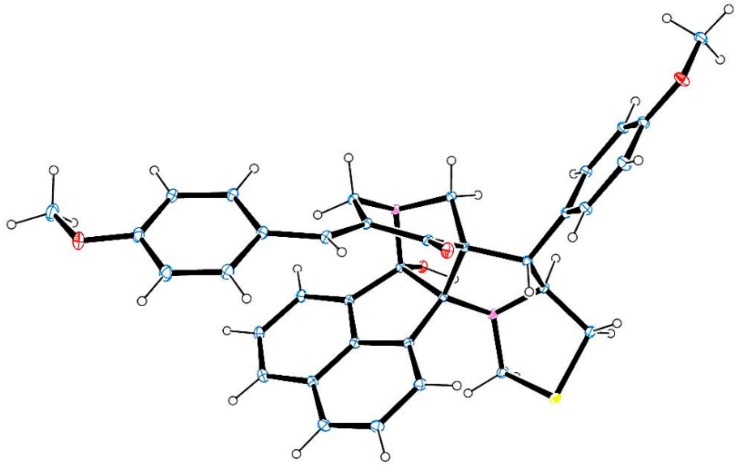
Oak Ridge Thermal Ellipsoid Plot (ORTEP) diagram of **4j**.

**Figure 2:**
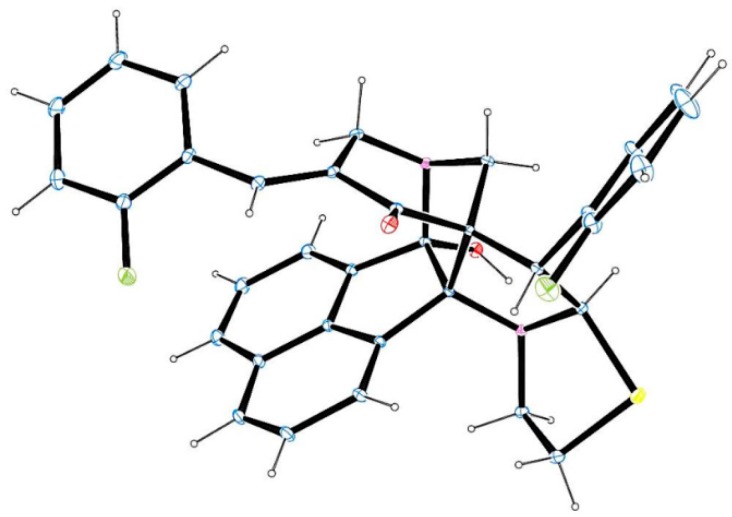
ORTEP diagram of **6f**.

The X-ray crystallographic study of a single crystal of **4j** (Figure 1) [33] and **6f** (Figure 2) [34] confirms the structure deduced from NMR spectroscopic studies.

**Scheme 4:**
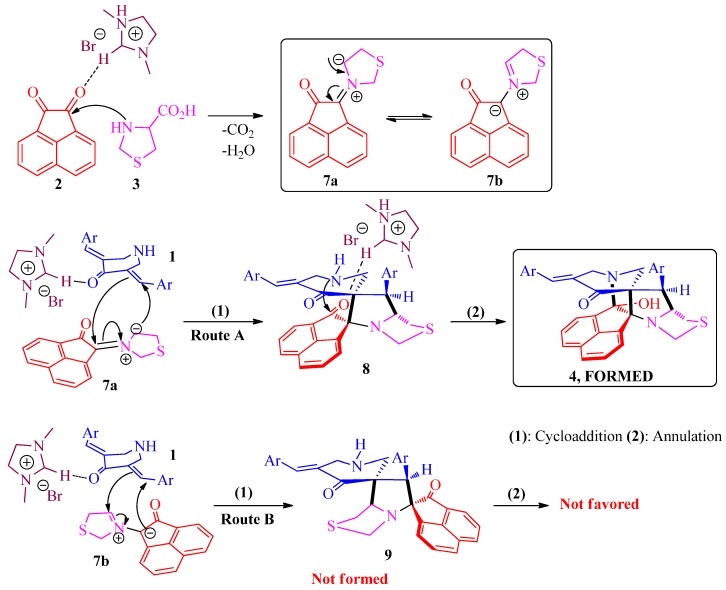
Probable mechanism for the formation of diazaheptacycles **4**.

A viable mechanism for the formation of diazaheptacycles **4** and **6** is shown in Scheme 4. Initially, the interaction of [bmim]Br with the carbonyl group of acenaphthenequinone **2** via hydrogen bonding would increase the electrophilicity of the carbonyl carbon, facilitating the nucleophilic attack of the NH of thiaproline **3**. Subsequent dehydration and concomitant decarboxylation furnishes azomethine ylide **7**, which may exist in the resonating forms **7a** and **7b** [35]. The interaction of [bmim]Br with the carbonyl group of 3,5-bis[(*E*)-arylmethylidene]tetrahydro-4(1*H*)-pyridinones **1** presumably activates the exocyclic double bond, allowing the initial addition reaction with the azomethine ylide that, in principle, can take place via reaction of **7a** (route A) or **7b** (route B) with the more electron deficient β-carbon of **1** to afford the spiropyrrolothiazoles **8** or **9**, respectively. However, the exclusive formation of **4** in the above reaction proves the selective cycloaddition of **7a** with **1** via route A to form **8**. Subsequently, the interaction of [bmim]Br with the second carbonyl group of the acenaphthenequinone ring of spiropyrrolothiazole **8** presumably increases the electrophilicity of that carbonyl carbon, facilitating further annulation by the reaction of amino function of piperidone ring with the proximate carbonyl group resulting in the formation of the cage framework **4**. In addition, the cycloaddition via route B is also ruled out from the fact that the dispiro intermediate **9** may not favor the subsequent annulation step to afford cage-like compounds in view of the higher distance between the reacting groups in **9**.

## 3. Experimental Section

### 3.1. General Methods

Melting points were taken using open capillary tubes and are uncorrected. ^1^H, ^13^C and two-dimensional NMR spectra were recorded on a Bruker 400 or 300 MHz instruments (Faellanden, Switzerland) in CDCl_3_ using Tetramethylsilane (TMS) as internal standard. Standard Bruker software was used throughout. Chemical shifts are given in parts per million (δ-scale) and the coupling constants are given in Hertz. IR spectra were recorded on a Perkin Elmer system 2000 FT-IR instrument (KBr) (Shelton, AL, USA). Single crystal X-ray data set for **4j** and **6f** was collected on Bruker APEXII DUO CCD area detector diffractometer (Karlsruhe, Germany) with Mo K_α_ (λ = 0.71073 Å) radiation. Elemental analyses were performed on a Perkin Elmer 2400 Series II Elemental CHNS analyzer (Waltham, MA, USA).

### 3.2. General Procedure for the Synthesis of Diazaheptacyclic Cage Compounds ***4a**–**n*** or ***6a**–**m***

An equimolar mixture of 3,5-bis[(*E*)-arylmethylidene]tetrahydro-4(1*H*)-pyridinone **1**, acenaphthenequinone **2** and thiaproline **3** or **5** in 100 mg of [bmim]Br was irradiated in a CEM microwave synthesizer at 100 °C for 4–8 min. After completion of the reaction (TLC), ethyl acetate (10 mL) was added and the reaction mixture stirred for 15 min. The ethyl acetate layer was then separated, washed with water (50 mL) and the solvent evaporated under reduced pressure. The resultant precipitate was dried in vacuum and subjected to crystallization from petroleum ether–ethyl acetate mixture (2:8) to obtain pure **4** or **6**. The ionic liquid [bmim]Br after extraction of the product was dried under vacuum and reused for subsequent reactions.

*14-Hydroxy-8-(phenyl)-11-[(E)-phenylmethylidene]-5-thia-3,13-diazaheptacyclo-[13.7.1.1^9,13^.0^2,9^.0^2,14^.0^3,7^.0^19,23^]tetracosa-1(22),15(23),16,18,20-pentaen-10-one* (**4a**) White solid, 92% (0.175 g), m.p. 140–142 °C, IR (KBr) υ_max_ 3420, 1721, 1685, 1602 cm^−1^; ^1^H-NMR (300 MHz, CDCl_3_): δ_H_ 3.03–3.12 (m, 2H, H-4a and H-6a), 3.22 (dd, *J* = 12.0, 6.3 Hz, 1H, H-6b), 3.35 (d, *J* = 17.4 Hz, 1H, H-12a), 3.44 (d, *J* = 11.4 Hz, 1H, H-24a), 3.68 (dd, *J* = 17.4, 1.8 Hz, 1H, H-12b), 3.81 (d, *J* = 11.4 Hz, 1H, H-24b), 4.27–4.35 (m, 2H, H-4b and H-8), 4.67–4.73 (m, 1H, H-7), 6.27 (s, 1H, H-25), 6.39–6.42 (m, 2H, ArH), 7.06–7.11 (m, 3H, ArH), 7.25–7.38 (m, 4H, ArH), 7.50–7.60 (m, 5H, ArH), 7.74 (d, *J* = 8.1 Hz, 1H, ArH), 7.83 (d, *J* = 6.9 Hz, 1H, ArH); ^13^C-NMR (75 MHz, CDCl_3_): δc 38.1, 51.4, 53.4, 56.4, 57.0, 72.7, 73.2, 96.3, 104.1, 121.6, 125.2, 126.2, 126.8, 127.8, 128.0, 128.2, 128.4, 128.8, 129.1, 129.6, 130.0, 131.2, 133.2, 134.2, 134.5, 136.2, 136.9, 137.0, 138.5, 196.7. Anal. calcd for C_34_H_28_N_2_O_2_S: C, 77.24; H, 5.34; N, 5.30. Found: C, 77.39; H, 5.23; N, 5.38%.

*14-Hydroxy-8-(2-methylphenyl)-11-[(E)-(2-methylphenyl)methylidene]-5-thia-3,13-diazaheptacyclo[13.7.1.1^9,13^.0^2,9^.0^2,14^.0^3,7^.0^19,23^]tetracosa-1(22),15(23),16,18,20-pentaen-10-one* (**4b**) Pale yellow solid, 90% (0.165 g), m.p. 127–129 °C, IR (KBr) υ_max_ 3422, 1725, 1680, 1598 cm^−1^; ^1^H-NMR (300 MHz, CDCl_3_): δ_H_ 1.56 (s, 3H, CH_3_), 2.82 (s, 3H, CH_3_), 2.98 (d, *J* = 12.3 Hz, 1H, H-6a), 3.14 (dd, *J* = 12.3, 6.3 Hz, 1H, H-6b), 3.32–3.44 (m, 3H, H-4a, H-12a and H-24a), 3.72 (d, *J* = 17.4 Hz, 1H, H-12b), 3.83 (d, *J* = 11.4 Hz, 1H, H-24b), 4.47–4.65 (m, 3H, H-4b and H-8 and H-7), 6.03 (d, *J* = 7.5 Hz, 1H, ArH), 6.53 (s, 1H, H-25), 6.84–7.04 (m, 3H, ArH), 7.18–7.45 (m, 5H, ArH), 7.57–7.69 (m, 3H, ArH), 7.78 (d, *J* = 8.1 Hz, 1H, ArH), 7.89 (d, *J* = 6.9 Hz, 1H, ArH); ^13^C-NMR (75 MHz, CDCl_3_): δc 19.6, 21.2, 37.2, 46.3, 52.8, 56.6, 57.4, 73.9, 74.9, 96.6, 103.8, 121.8, 125.1, 125.4, 126.1, 126.3, 126.8, 126.9, 127.7, 128.2, 128.4, 128.5, 128.9, 130.1, 131.3, 131.8, 132.7, 133.4, 134.4, 135.4, 135.8, 137.1, 137.5, 138.7, 139.3, 196.2. Anal. calcd for C_36_H_32_N_2_O_2_S: C, 77.67; H, 5.79; N, 5.03. Found: C, 77.80; H, 5.70; N, 5.12%.

*14-Hydroxy-8-(2-methoxylphenyl)-11-[(E)-(2-methoxylphenyl)methylidene]-5-thia-3,13-diazaheptacyclo[13.7.1.1^9,13^.0^2,9^.0^2,14^.0^3,7^.0^19,23^]tetracosa-1(22),15(23),16,18,20-pentaen-10-one* (**4c**) Pale yellow solid, 85% (0.149 g), m.p. 166–168 °C, IR (KBr) υ_max_ 3426, 1722, 1683, 1600 cm^−1^; ^1^H-NMR (300 MHz, CDCl_3_): δ_H_ 3.03 (d, *J* = 12.0 Hz, 1H, H-6a), 3.12 (dd, *J* = 12.0, 6.3 Hz, 1H, H-6b), 3.28–3.40 (m, 3H, H-4a, H-12a and H-24a), 3.56 (s, 3H, OCH_3_), 3.68 (dd, *J* = 17.7, 2.1 Hz, 1H, H-12b), 3.81 (d, *J* = 11.1 Hz, 1H, H-24b), 3.96 (s, 3H, OCH_3_), 4.59–4.83 (m, 3H, H-4b and H-8 and H-7), 5.99 (dd, *J* = 7.8, 1.2 Hz, 1H, ArH), 6.47 (s, 1H, H-25), 6.57–6.63 (m, 2H, ArH), 6.93–7.11 (m, 3H, ArH), 7.22–7.37 (m, 2H, ArH), 7.48 (dd, *J* = 8.1, 1.8 Hz, 1H, ArH), 7.55–7.60 (m, 3H, ArH), 7.74 (d, *J* = 8.1 Hz, 1H, ArH), 7.90 (d, *J* = 6.9 Hz, 1H, ArH); ^13^C-NMR (75 MHz, CDCl_3_): δc 37.6, 47.1, 53.1, 55.2, 56.2, 56.3, 57.6, 71.8, 73.8, 96.0, 103.5, 110.3, 112.0, 119.8, 121.1, 121.4, 123.4, 125.2, 125.5, 126.0, 126.6, 127.8, 128.3, 129.1, 129.9, 130.4, 131.3, 131.8, 131.9, 133.0, 134.5, 137.1, 138.7, 157.7, 158.9, 196.5. Anal. calcd for C_36_H_32_N_2_O_4_S: C, 73.45; H, 5.48; N, 4.76. Found: C, 73.63; H, 5.39; N, 4.89%.

*8-(2-Bromophenyl)-11-[(E)-(2-bromophenyl)methylidene]-14-hydroxy-5-thia-3,13-diazaheptacyclo[13.7.1.1^9,13^.0^2,9^.0^2,14^.0^3,7^.0^19,23^]tetracosa-1(22),15(23),16,18,20-pentaen-10-one* (**4d**) White solid, 91% (0.144 g), m.p. 184–186 °C, IR (KBr) υ_max_ 3424, 1725, 1680, 1595 cm^−1^; ^1^H-NMR (300 MHz, CDCl_3_): δ_H_ 3.11 (dd, *J* = 12.3, 6.3 Hz, 1H, H-6b), 3.20 (d, *J* = 12.3 Hz, 1H, H-6a), 3.36–3.42 (m, 3H, H-4a, H-12a and H-24a), 3.68 (dd, *J* = 17.7, 3.0 Hz, 1H, H-12b), 3.82 (d, *J* = 11.4 Hz, 1H, H-24b), 4.37–5.07 (m, 3H, H-4b and H-8 and H-7), 5.82–5.87 (m, 1H, ArH), 6.51 (s, 1H, H-25), 6.93–7.00 (m, 2H, ArH), 7.12–7.18 (m, 1H, ArH), 7.27–7.50 (m, 4H, ArH), 7.57–7.76 (m, 4H, ArH), 7.82 (d, *J* = 8.1 Hz, 1H, ArH), 7.96 (d, *J* = 6.9 Hz, 1H, ArH); ^13^C-NMR (75 MHz, CDCl_3_): δc 36.6, 49.3, 52.4, 56.3, 57.2, 74.0, 74.7, 96.3, 103.5, 121.6, 124.2, 125.4, 126.5, 126.9, 127.4, 127.7, 127.9, 128.2, 128.3, 128.7, 129.3, 129.5, 130.0, 131.5, 132.9, 133.7, 133.9, 134.6, 134.7, 135.3, 137.0, 137.1, 138.5, 195.3. Anal. calcd for C_34_H_26_Br_2_N_2_O_2_S: C, 59.49; H, 3.82; N, 4.08. Found: C, 59.64; H, 3.70; N, 4.21%.

*8-(2-Chlorophenyl)-11-[(E)-(2-chlorophenyl)methylidene]-14-hydroxy-5-thia-3,13-diazaheptacyclo[13.7.1.1^9,13^.0^2,9^.0^2,14^.0^3,7^.0^19,23^]tetracosa-1(22),15(23),16,18,20-pentaen-10-one* (**4e**) White solid, 90% (0.156 g), m.p. 146–148 °C, IR (KBr) υ_max_ 3418, 1720, 1681, 1601 cm^−1^; ^1^H-NMR (300 MHz, CDCl_3_): δ_H_ 3.12–3.14 (m, 2H, H-6a and H-6b), 3.34–3.42 (m, 3H, H-4a, H-12a and H-24a), 3.69 (dd, *J* = 17.7, 2.7 Hz, 1H, H-12b), 3.81 (d, *J* = 11.4 Hz, 1H, H-24b), 4.39–5.06 (m, 3H, H-4b and H-8 and H-7), 5.98 (d, *J* = 7.5 Hz, 1H, ArH), 6.51 (s, 1H, H-25), 6.90–7.13 (m, 3H, ArH), 7.19–7.29 (m, 2H, ArH), 7.37–7.62 (m, 5H, ArH), 7.69 (d, *J* = 8.1 Hz, 1H, ArH), 7.80 (d, *J* = 8.4 Hz, 1H, ArH), 7.95 (d, *J* = 6.9 Hz, 1H, ArH); ^13^C-NMR (75 MHz, CDCl_3_): δc 36.9, 46.5, 52.7, 56.3, 57.4, 74.0, 74.4, 96.3, 103.6, 121.6, 125.3, 126.2, 126.5, 127.3, 127.4, 128.0, 128.3, 128.7, 129.0, 129.7, 130.0, 131.2, 131.4, 132.7, 132.9, 133.7, 134.2, 134.4, 135.3, 136.6, 137.0, 138.4, 195.5. Anal. calcd for C_34_H_26_Cl_2_N_2_O_2_S: C, 68.34; H, 4.39; N, 4.69. Found: C, 68.50; H, 4.48; N, 4.52%.

*8-(2-Fluorophenyl)-11-[(E)-(2-fluorophenyl)methylidene]-14-hydroxy-5-thia-3,13-diazaheptacyclo[13.7.1.1^9,13^.0^2,9^.0^2,14^.0^3,7^.0^19,23^]tetracosa-1(22),15(23),16,18,20-pentaen-10-one* (**4f**) Light brown solid, 88% (0.160 g), m.p. 150–152 °C, IR (KBr) υ_max_ 3419, 1724, 1680, 1598 cm^−1^; ^1^H-NMR (300 MHz, CDCl_3_): δ_H_ 3.02 (d, *J* = 12.3 Hz, 1H, H-6a), 3.16–3.31 (m, 3H, H-4a, H-6b and H-12a), 3.43 (d, *J* = 11.1 Hz, 1H, H-24a), 3.67 (dd, *J* = 17.7, 1.8 Hz, 1H, H-12b), 3.82 (d, *J* = 11.4 Hz, 1H, H-24b), 4.46–4.77 (m, 3H, H-4b and H-8 and H-7), 6.12–6.22 (m, 3H, H-25 and ArH), 6.76–6.86 (m, 2H, ArH), 7.08–7.31 (m, 4H, ArH), 7.51–7.62 (m, 4H, ArH), 7.79 (d, *J* = 8.4 Hz, 1H, ArH), 7.88 (d, *J* = 6.9 Hz, 1H, ArH); ^13^C-NMR (75 MHz, CDCl_3_): δc 37.7, 46.3, 53.3, 56.3, 57.3, 71.6, 73.8, 96.0, 103.7, 115.5, 116.8, 121.5, 122.1, 123.6, 123.8, 124.7, 125.2, 126.2, 127.1, 127.8, 128.2, 128.5, 129.7, 130.5, 130.9, 131.2, 131.9, 134.1, 135.1, 137.0, 138.4, 159.5, 162.8, 196.2. Anal. calcd for C_34_H_26_F_2_N_2_O_2_S: C, 72.32; H, 4.64; N, 4.96. Found: C, 72.20; H, 4.75; N, 4.80%.

*14-Hydroxy-8-(3-nitrophenyl)-11-[(E)-(3-nitrophenyl)methylidene]-5-thia-3,13-diazaheptacyclo[13.7.1.1^9,13^.0^2,9^.0^2,14^.0^3,7^.0^19,23^]tetracosa-1(22),15(23),16,18,20-pentaen-10-one* (**4g**) Dark brown solid, 91% (0.154 g), m.p. 176–178 °C, IR (KBr) υ_max_ 3424, 1716, 1686, 1616 cm^−1^; ^1^H-NMR (400 MHz, CDCl_3_): δ_H_ 3.02–3.09 (m, 2H, H-4a and H-6a), 3.23 (d, *J* = 17.6Hz, 1H, H-12a), 3.28 (dd, *J* = 12.4, 6.4 Hz, 1H, H-6b), 3.48 (d, *J* = 11.6 Hz, 1H, H-24a), 3.65 (dd, *J* = 17.6, 2.8 Hz, 1H, H-12b), 3.85 (d, *J* = 11.6 Hz, 1H, H-24b), 4.37–4.40 (m, 2H, H-4b and H-8), 4.75–4.80 (m, 1H, H-7), 6.16 (s, 1H, H-25), 6.69 (d, *J* = 8.0 Hz, 1H, ArH), 7.12 (s, 1H, ArH), 7.24–7.30 (m, 2H, ArH), 7.50–7.51 (m, 3H, ArH), 7.65–7.69 (m, 1H, ArH), 7.84–7.88 (m, 2H, ArH), 7.95 (d, *J* = 7.6 Hz, 1H, ArH), 7.99 (dd, *J* = 8.0, 1.6 Hz, 1H, ArH), 8.18 (dd, *J* = 7.2, 1.2 Hz, 1H, ArH), 8.46–8.48 (m, 1H, ArH); ^13^C-NMR (100 MHz, CDCl_3_): δc 37.9, 50.9, 53.2, 56.4, 56.9, 72.2, 73.5, 96.3, 104.1, 121.8, 123.2, 123.3, 124.2, 124.5, 125.2, 126.2, 127.2, 128.1, 128.7, 129.2, 130.2, 131.3, 133.1, 134.1, 134.9, 135.1, 135.9, 136.0, 136.9, 138.2, 139.0, 148.0, 148.9, 196.4. Anal. calcd for C_34_H_26_N_4_O_6_S: C, 66.01; H, 4.24; N, 9.06. Found: C, 66.19; H, 4.45; N, 9.19%.

*8-(2,4-Dichlorophenyl)-11-[(E)-(2,4-dichlorophenyl)methylidene]-14-hydroxy-5-thia-3,13-diazaheptacyclo[13.7.1.1^9,13^.0^2,9^.0^2,14^.0^3,7^.0^19,23^]tetracosa-1(22),15(23),16,18,20-pentaen-10-one* (**4h**) Light brown solid, 90% (0.145 g), m.p. 144–146 °C, IR (KBr) υ_max_ 3409, 1686, 1605 cm^−1^; ^1^H-NMR (300 MHz, CDCl_3_): δ_H_ 3.07–3.16 (m, 2H, H-4a and H-6a), 3.27–3.49 (m, 3H, H-6b, H-12a and H-24a), 3.67 (dd, *J* = 18.0, 3.0 Hz, 1H, H-12b), 3.82 (d, *J* = 11.4 Hz, 1H, H-24b), 4.36–4.99 (m, 3H, H-4b, H-7 and H-8), 5.92 (d, *J* = 8.4 Hz, 1H, ArH), 6.40 (s, 1H, H-25), 6.93 (dd, *J* = 8.4, 2.1 Hz, 1H, ArH), 7.15–7.48 (m, 3H, ArH), 7.43–7.48 (m, 1H, ArH), 7.54–7.64 (m, 3H, ArH), 7.71 (d, *J* = 8.1 Hz, 1H, ArH), 7.81 (d, *J* = 8.1 Hz, 1H, ArH), 7.91 (m, *J* = 6.6 Hz, 1H, ArH); ^13^C-NMR (75 MHz, CDCl_3_): δc 36.8, 46.1, 52.6, 56.3, 57.2, 74.0, 74.2, 96.2, 103.6, 121.6, 125.3, 126.5, 126.7, 127.4,127.6, 128.2,128.7, 129.1, 129.6, 130.3, 131.0, 131.1, 131.3, 131.7, 133.5, 133.8, 134.3, 134.6, 135.1, 135.2, 136.9, 137.3, 138.3, 195.3. Anal. calcd for C_34_H_24_Cl_4_N_2_O_2_S: C, 61.28; H, 3.63; N, 4.20. Found: C, 61.46; H, 3.49; N, 4.33%.

*14-Hydroxy-8-(4-methylphenyl)-11-[(E)-(4-methylphenyl)methylidene]-5-thia-3,13-diazaheptacyclo[13.7.1.1^9,13^.0^2,9^.0^2,14^.0^3,7^.0^19,23^]tetracosa-1(22),15(23),16,18,20-pentaen-10-one* (**4i**) Light brown solid, 93% (0.170 g), m.p. 141–143 °C, IR (KBr) υ_max_ 3416, 1723, 1682, 1594 cm^−1^; ^1^H-NMR (400 MHz, CDCl_3_): δ_H_ 2.23 (s, 3H, CH_3_), 2.33 (s, 3H, CH_3_), 3.03–3.09 (m, 2H, H-4a and H-6a), 3.21 (dd, *J* = 12.0, 6.4 Hz, 1H, H-6b), 3.34 (d, *J* = 17.6 Hz, 1H, H-12a), 3.43 (d, *J* = 11.2 Hz, 1H, H-24a), 3.68 (dd, *J* = 17.6, 2.0 Hz, 1H, H-12b), 3.81 (d, *J* = 11.2 Hz, 1H, H-24b), 4.24–4.31 (m, 2H, H-4b and H-8), 4.65–4.69 (m, 1H, H-7), 6.29 (s, 1H, H-25), 6.35 (d, *J* = 8.0 Hz, 2H, ArH), 6.88 (d, *J* = 8.0 Hz, 2H, ArH), 7.17 (d, *J* = 8.0 Hz, 2H, ArH), 7.30–7.34 (m, 1H, ArH), 7.43 (d, *J* = 8.0 Hz, 2H, ArH), 7.52–7.61 (m, 3H, ArH), 7.72 (d, *J* = 8.0 Hz, 1H, ArH), 7.82 (d, *J* = 6.8 Hz, 1H, ArH); ^13^C-NMR (100 MHz, CDCl_3_): δc 21.5, 21.7, 38.1, 51.0, 53.3, 56.4, 56.8, 72.7, 73.0, 96.2, 104.1, 121.5, 125.2, 126.3, 126.7, 127.9, 128.4, 129.0, 129.4, 129.8, 130.2, 131.2, 131.5, 132.1, 133.9, 134.5, 136.5, 137.0, 137.6, 138.5, 139.2, 196.4. Anal. calcd for C_36_H_32_N_2_O_2_S: C, 77.67; H, 5.79; N, 5.03. Found: C, 77.79; H, 5.68; N, 5.12%.

*14-Hydroxy-8-(4-methoxyphenyl)-11-[(E)-(4-methoxyphenyl)methylidene]-5-thia-3,13-diazaheptacyclo[13.7.1.1^9,13^.0^2,9^.0^2,14^.0^3,7^.0^19,23^]tetracosa-1(22),15(23),16,18,20-pentaen-10-one* (**4j**) Pale yellow solid, 87% (0.152 g), m.p. 137–139 °C, IR (KBr) υ_max_ 3422, 1720, 1684, 1600 cm^−1^; ^1^H-NMR (300 MHz, CDCl_3_): δ_H_ 3.03–3.11 (m, 2H, H-4a and H-6a), 3.22 (dd, *J* = 12.0, 6.6 Hz, 1H, H-6b), 3.34 (d, *J* = 17.1 Hz, 1H, H-12a), 3.42–3.49 (m, 1H, H-24a), 3.68–3.70 (m, 2H, H-12b and H-24b), 3.73 (s, 3H, OCH_3_), 3.79 (s, 3H, OCH_3_), 4.23–4.30 (m, 2H, H-4b and H-8), 4.63–4.68 (m, 1H, H-7), 6.27 (s, 1H, H-25), 6.47 (d, *J* = 8.7 Hz, 2H, ArH), 6.62 (d, *J* = 8.7 Hz, 2H, ArH), 6.89 (d, *J* = 8.7 Hz, 2H, ArH), 7.31–7.36 (m, 1H, ArH), 7.45–7.63 (m, 5H, ArH), 7.71 (d, *J* = 8.1 Hz, 1H, ArH), 7.82 (d, *J* = 6.9 Hz, 1H, ArH); ^13^C-NMR (75 MHz, CDCl_3_): δc 38.0, 50.6, 53.4, 55.6, 55.7, 56.4, 56.8, 72.7, 72.9, 96.1, 104.1, 113.8, 114.4, 121.5, 125.1, 126.2, 126.6, 127.1, 127.9, 128.3, 129.0, 130.5, 131.0, 131.2, 132.1, 134.7, 136.1, 137.0, 138.6, 159.3, 160.2, 196.7. Anal. calcd for C_36_H_32_N_2_O_4_S: C, 73.45; H, 5.48; N, 4.76. Found: C, 73.31; H, 5.34; N, 4.87%.

*8-(4-Bromophenyl)-11-[(E)-(4-bromophenyl)methylidene]-14-hydroxy-5-thia-3,13-diazaheptacyclo[13.7.1.1^9,13^.0^2,9^.0^2,14^.0^3,7^.0^19,23^]tetracosa-1(22),15(23),16,18,20-pentaen-10-one* (**4k**) Light brown solid, 92% (0.146 g), m.p. 171–173 °C, IR (KBr) υ_max_ 3418, 1724, 1680, 1597 cm^−1^; ^1^H-NMR (300 MHz, CDCl_3_): δ_H_ 3.00–3.06 (m, 2H, H-4a and H-6a), 3.20–3.30 (m, 1H, H-6b and H-12a), 3.44 (d, *J* = 11.4 Hz, 1H, H-24a), 3.63 (dd, *J* = 17.7, 2.1 Hz, 1H, H-12b), 3.81 (d, *J* = 11.4 Hz, 1H, H-24b), 4.22–4.29 (m, 2H, H-4b and H-8), 4.62–4.68 (m, 1H, H-7), 6.16 (s, 1H, H-25), 6.26 (d, *J* = 8.4 Hz, 2H, ArH), 7.21–7.60 (m, 10H, ArH), 7.75 (d, *J* = 8.1 Hz, 1H, ArH), 7.80 (d, *J* = 6.9 Hz, 1H, ArH); ^13^C-NMR (75 MHz, CDCl_3_): δc 38.0, 50.7, 53.2, 56.4, 56.8, 72.4, 73.1, 96.2, 104.1, 121.7, 122.1, 123.3, 125.1, 126.3, 126.9, 128.1, 128.5, 130.4, 131.2, 131.4, 131.5, 132.2, 133.0, 133.7, 134.9, 135.9, 135.4, 136.9, 138.3, 196.5. Anal. calcd for C_34_H_26_Br_2_N_2_O_2_S: C, 59.49; H, 3.82; N, 4.08. Found: C, 59.62; H, 3.90; N, 4.21%.

*8-(4-Chlorophenyl)-11-[(E)-(4-chlorophenyl)methylidene]-14-hydroxy-5-thia-3,13-diazaheptacyclo[13.7.1.1^9,13^.0^2,9^.0^2,14^.0^3,7^.0^19,23^]tetracosa-1(22),15(23),16,18,20-pentaen-10-one* (**4l**) Brown solid, 95% (0.165 g), m.p. 154–156 °C, IR (KBr) υ_max_ 3402, 1719, 1686, 1601 cm^−1^; ^1^H-NMR (400 MHz, CDCl_3_): δ_H_ 2.99–3.04 (m, 2H, H-4a and H-6a), 3.19–3.29 (m, 2H, H-6b and H-12a), 3.43 (d, *J* = 11.6 Hz, 1H, H-24a), 3.62 (dd, *J* = 17.6, 2.4 Hz, 1H, H-12b), 3.80 (d, *J* = 11.2 Hz, 1H, H-24b), 4.23–4.27 (m, 2H, H-4b and H-8), 4.61–4.66 (m, 1H, H-7), 6.17 (s, 1H, H-25), 6.32 (d, *J* = 8.4 Hz, 2H, ArH), 7.05 (d, *J* = 8.4 Hz, 2H, ArH), 7.27–7.35 (m, 3H, ArH), 7.48 (d, *J* = 8.4 Hz, 2H, ArH), 7.55–7.69 (m, 3H, ArH), 7.73 (d, *J* = 8.4 Hz, 1H, ArH), 7.79 (d, *J* = 6.8 Hz, 1H, ArH); ^13^C-NMR (100 MHz, CDCl_3_): δc 38.0, 50.7, 53.2, 56.4, 56.8, 72.4, 73.1, 96.2, 104.1, 121.7, 125.1, 126.3, 126.9, 128.1, 128.4, 128.5, 128.8, 129.3, 130.9, 131.2, 132.5, 133.6, 133.9, 134.3, 134.8, 134.9, 135.4, 136.9, 138.3, 196.5. Anal. calcd for C_34_H_26_Cl_2_N_2_O_2_S: C, 68.34; H, 4.39; N, 4.69. Found: C, 68.45; H, 4.51; N, 4.78%.

*8-(4-Fluorophenyl)-11-[(E)-(4-fluorophenyl)methylidene]-14-hydroxy-5-thia-3,13-diazaheptacyclo[13.7.1.1^9,13^.0^2,9^.0^2,14^.0^3,7^.0^19,23^]tetracosa-1(22),15(23),16,18,20-pentaen-10-one* (**4m**) White solid, 93% (0.169 g), m.p. 132–134 °C, IR (KBr) υ_max_ 3416, 1682, 1601 cm^−1^; ^1^H-NMR (400 MHz, CDCl_3_): δ_H_ 3.02–3.09 (m, 2H, H-4a and H-6a), 3.21–3.26 (m, 1H, H-6b), 3.30 (d, *J* = 17.6 Hz, 1H, H-12a), 3.45 (d, *J* = 11.2 Hz, 1H, H-24a), 3.67 (dd, *J* = 17.2, 2.0 Hz, 1H, H-12b), 3.82 (d, *J* = 11.6 Hz, 1H, H-24b), 4.25–4.31 (m, 2H, H-4b and H-8), 4.64–4.68 (m, 1H, H-7), 6.20 (s, 1H, H-25), 6.39–6.42 (m, 2H, ArH), 6.76–6.81 (m, 2H, ArH), 7.04–7.08 (m, 2H, ArH), 7.31–7.34 (m, 1H, ArH), 7.51–7.56 (m, 3H, ArH), 7.59 (d, *J* = 6.8 Hz, 2H, ArH), 7.74 (d, *J* = 8.4 Hz, 1H, ArH), 7.81 (d, *J* = 7.2 Hz, 1H, ArH); ^13^C-NMR (100 MHz, CDCl_3_): δ_C_ 38.0, 50.6, 53.3, 56.4, 56.9, 72.6, 73.1, 96.2, 104.1, 115.4, 116.0, 121.6, 125.1, 126.2, 126.8, 128.0, 128.4, 130.3, 131.0, 131.2, 131.9, 132.5, 133.0, 134.5, 135.0, 137.0,138.4, 161.5,163.9, 196.7. Anal. calcd for C_34_H_26_F_2_N_2_O_2_S: C, 72.32; H, 4.64; N, 4.96. Found: C, 73.20; H, 4.79; N, 4.78%.

*14-Hydroxy-8-(naphthyl)-11-[(E)-naphthylmethylidene]-5-thia-3,13-diazaheptacyclo-[13.7.1.1^9,13^.0^2,9^.0^2,14^.0^3,7^.0^19,23^]tetracosa-1(22),15(23),16,18,20-pentaen-10-one* (**4n**) White solid, 89% (0.149 g), m.p. 158–160 °C, IR (KBr) υ_max_ 3423, 1721, 1684, 1590 cm^−1^; ^1^H-NMR (300 MHz, CDCl_3_): δ_H_ 2.96 (d, *J* = 12.3 Hz, 1H, H-6a), 3.13 (dd, *J* = 12.3, 6.3 Hz, 1H, H-6b), 3.30 (d, *J* = 18.0 Hz, 1H, H-12a), 3.41–3.52 (m, 2H, H-4a and H-24a), 3.73 (dd, *J* = 18.0, 2.1 Hz, 1H, H-12b), 3.88 (d, *J* = 11.4 Hz, 1H, H-24b), 4.66–4.77 (m, 2H, H-4b and H-7), 5.32 (d, *J* = 10.2 Hz, 1H, H-8), 6.22 (d, *J* = 6.9 Hz, 1H, ArH), 6.69 (d, *J* = 8.4 Hz, 1H, ArH), 6.93 (s, 1H, H-25), 7.11–7.91 (m, 16H, ArH), 8.15 (d, *J* = 6.9 Hz, 1H, ArH), 8.92 (d, *J* = 8.7 Hz, 1H, ArH); ^13^C-NMR (75 MHz, CDCl_3_): δc 37.6, 44.8, 53.2, 56.6, 57.8, 73.8, 75.5, 97.0, 104.0, 121.6, 124.8, 125.0, 125.2, 125.4, 126.1, 126.3, 126.4, 127.0, 127.1, 128.1, 128.4, 128.5, 128.8, 129.2, 129.4, 131.2, 131.3, 131.4, 133.3, 133.6, 134.0, 134.1, 134.4, 134.7, 134.8, 137.2, 138.5, 196.4. Anal. calcd for C_42_H_32_N_2_O_2_S: C, 80.23; H, 5.13; N, 4.46. Found: C, 80.38; H, 5.29; N, 4.35%.

*14-Hydroxy-8-(phenyl)-11-[(E)-phenylmethylidene]-6-thia-3,13-diazaheptacyclo-[13.7.1.1^9,13^.0^2,9^.0^2,14^.0^3,7^.0^19,23^]tetracosa-1(22),15(23),16,18,20-pentaen-10-one* (**6a**) White solid, 93% (0.178 g), m.p. 135–137 °C, IR (KBr) υ_max_ 3418, 1721, 1692, 1599 cm^−1^; ^1^H-NMR (300 MHz, CDCl_3_): δ_H_ 2.79–2.99 (m, 5H, H-4a, H-4b, H-5a, H-24a and H-24b), 3.40 (d, *J* = 17.4 Hz, 1H, H-12a), 3.67 (dd, *J* = 17.4, 2.7 Hz, 1H, H-12b), 4.25 (dd, *J* = 12.6, 2.1 Hz, 1H, H-5b), 4.91 (d, *J* = 6.9 Hz, 1H, H-8), 5.70 (d*, J* = 7.2 Hz, 1H, H-7), 5.94 (brs, 1H, OH), 6.28 (s, 1H, H-25), 6.40–6.43 (m, 2H, ArH), 7.05–7.15 (m, 3H, ArH), 7.25–7.39 (m, 4H, ArH), 7.51–7.62 (m, 6H, ArH), 7.74 (dd, *J* = 6.9, 2.1 Hz, 1H, ArH); ^13^C-NMR (75 MHz, CDCl_3_): δc 33.9, 53.1, 53.5, 53.9, 57.6, 75.2, 78.0, 95.1, 103.8, 121.7, 124.2, 126.3, 126.8, 127.8, 128.1, 128.2, 128.5, 128.9, 129.0, 129.1, 130.0, 131.2, 133.3, 134.1, 136.0, 136.5, 137.2, 137.3, 138.2, 196.4. Anal. calcd for C_34_H_28_N_2_O_2_S: C, 77.24; H, 5.34; N, 5.30. Found: C, 77.43; H, 5.20; N, 5.17%.

*14-Hydroxy-8-(2-methylphenyl)-11-[(E)-(2-methylphenyl)methylidene]-6-thia-3,13-diazaheptacyclo[13.7.1.1^9,13^.0^2,9^.0^2,14^.0^3,7^.0^19,23^]tetracosa-1(22),15(23),16,18,20-pentaen-10-one* (**6b**) Pale yellow solid, 90% (0.165 g), m.p. 179–181 °C, IR (KBr) υ_max_ 3398, 1719, 1680, 1598 cm^−1^; ^1^H-NMR (300 MHz, CDCl_3_): δ_H_ 1.56 (s, 3H, CH_3_), 2.79–2.96 (m, 7H, CH_3_, H-4a, H-4b, H-24a and H-24b), 3.23 (d, *J* = 12.6 Hz, 1H, H-5a), 3.38 (d, *J* = 18.0 Hz, 1H, H-12a), 3.77 (dd, *J* = 17.7, 2.7 Hz, 1H, H-12b), 4.63 (d, *J* = 12.6 Hz, 1H, H-5b), 5.07 (d, *J* = 7.8 Hz, 1H, H-8), 5.46 (d, *J* = 8.1 Hz, 1H, H-7), 6.06 (d, *J* = 7.5 Hz, 1H, ArH), 6.55 (s, 1H, H-25), 6.84–6.91 (m, 2H, ArH), 7.00 (d, *J* = 7.2 Hz, 1H, ArH), 7.15–7.26 (m, 3H, ArH), 7.39 (d, *J* = 6.9 Hz, 1H, ArH), 7.44 (d, *J* = 7.8 Hz, 1H, ArH), 7.56–7.62 (m, 3H, ArH), 7.67 (d, *J* = 8.1 Hz, 1H, ArH), 7.78 (dd, *J* = 6.3, 2.7 Hz, 1H, ArH); ^13^C-NMR (75 MHz, CDCl_3_): δc 19.6, 21.0, 33.4, 50.4, 52.8, 53.1, 57.7, 75.7, 80.3, 95.5, 103.6, 121.9, 124.1, 125.4, 126.2, 126.6, 126.8, 127.4, 127.7, 128.3, 128.5, 128.6, 129.0, 130.1, 131.3, 131.7, 132.7, 133.3, 135.7, 135.9, 136.0, 137.3, 137.6, 138.5, 139.1, 195.8. Anal. calcd for C_36_H_32_N_2_O_2_S: C, 77.67; H, 5.79; N, 5.03. Found: C, 77.51; H, 5.91; N, 5.15%.

*14-Hydroxy-8-(2-methoxylphenyl)-11-[(E)-(2-methoxylphenyl)methylidene]-6-thia-3,13-diazaheptacyclo[13.7.1.1^9,13^.0^2,9^.0^2,14^.0^3,7^.0^19,23^]tetracosa-1(22),15(23),16,18,20-pentaen-10-one* (**6c**) White solid, 84% (0.147 g), m.p. 175–177 °C, IR (KBr) υ_max_ 3416, 1721, 1681, 1601 cm^−1^; ^1^H-NMR (300 MHz, CDCl3): δ_H_ 2.72–2.97 (m, 4H, H-4a, H-4b, H-24a and H-24b), 3.09 (d, *J* = 12.6 Hz, 1H, H-5a), 3.26 (d, *J* = 17.4 Hz, 1H, H-12a), 3.56 (s, 3H, OCH_3_), 3.62 (d, *J* = 17.4 Hz, 1H, H-12b), 3.92 (s, 3H, OCH_3_), 4.62 (d, *J* = 12.6 Hz, 1H, H-5b), 5.05 (d, *J* = 6.9 Hz, 1H, H-8), 5.96 (d, *J* = 6.9 Hz, 1H, H-7), 6.01 (d, *J* = 7.5 Hz, 1H, ArH), 6.51 (s, 1H, H-25), 6.55–6.62 (m, 2H, ArH), 6.90–7.59 (m, 9H, ArH), 7.66 (d, *J* = 6.6 Hz, 1H, ArH), 7.73 (d, *J* = 8.1 Hz, 1H, ArH); ^13^C-NMR (75 MHz, CDCl_3_): δc 33.7, 51.8, 52.9, 53.1, 55.2, 55.9, 57.5, 75.7, 76.9, 94.6, 103.5, 110.3, 111.9, 119.8, 121.2, 121.5, 123.3, 124.4, 125.2, 126.1, 126.5, 128.0, 128.4, 129.1, 129.9, 130.5, 131.2, 132.2, 133.0, 136.3, 137.3, 138.5, 157.7, 158.5, 195.9. Anal. calcd for C_36_H_32_N_2_O_4_S: C, 73.45; H, 5.48; N, 4.76. Found: C, 73.60; H, 5.32; N, 4.68%.

*8-(2-Bromophenyl)-11-[(E)-(2-bromophenyl)methylidene]-14-hydroxy-6-thia-3,13-diazaheptacyclo[13.7.1.1^9,13^.0^2,9^.0^2,14^.0^3,7^.0^19,23^]tetracosa-1(22),15(23),16,18,20-pentaen-10-one* (**6d**) White solid, 92% (0.146 g), m.p. 172–174 °C, IR (KBr) υ_max_ 3396, 1718, 1681, 1602 cm^−1^; ^1^H-NMR (300 MHz, CDCl3): δ_H_ 2.83–2.99 (m, 4H, H-4a, H-4b, H-24a and H-24b), 3.29 (d, *J* = 12.3 Hz, 1H, H-5a), 3.40 (d, *J* = 17.7 Hz, 1H, H-12a), 3.68 (dd, *J* = 17.7, 2.7 Hz, 1H, H-12b), 4.65 (d, *J* = 12.3 Hz, 1H, H-5b), 5.37 (d, *J* = 8.4 Hz, 1H, H-8), 5.41 (d, *J* = 8.7 Hz, 1H, H-7), 5.91–5.94 (m, 1H, ArH), 6.11 (brs, 1H, OH), 6.54 (s, 1H, H-25), 6.96–6.99 (m, 2H, ArH), 7.11–7.78 (m, 10H, ArH), 7.82 (d, *J* = 7.5 Hz, 1H, ArH); ^13^C-NMR (75 MHz, CDCl_3_): δc 33.4, 52.4, 53.1, 53.4, 57.4, 75.6, 79.2, 95.0, 103.4, 121.7, 124.1, 124.3, 126.6, 126.9, 127.2, 127.4, 127.8, 128.4, 128.6, 128.7, 129.3, 129.6, 130.1, 131.5, 133.0, 133.8, 134.5, 134.6, 135.2, 135.7, 136.7, 137.1, 138.3, 194.8. Anal. calcd for C_34_H_26_Br_2_N_2_O_2_S: C, 59.49; H, 3.82; N, 4.08. Found: C, 59.32; H, 3.73; N, 4.17%.

*8-(2-Chlorophenyl)-11-[(E)-(2-chlorophenyl)methylidene]-14-hydroxy-6-thia-3,13-diazaheptacyclo[13.7.1.1^9,13^.0^2,9^.0^2,14^.0^3,7^.0^19,23^]tetracosa-1(22),15(23),16,18,20-pentaen-10-one* (**6e**) White solid, 87% (0.150 g), m.p. 134–136 °C, IR (KBr) υ_max_ 3398, 1725, 1685, 1603 cm^−1^; ^1^H-NMR (300 MHz, CDCl3): δ_H_ 2.83–3.02 (m, 4H, H-4a, H-4b, H-24a and H-24b), 3.25 (d, *J* = 12.3 Hz, 1H, H-5a), 3.37 (d, *J* = 17.7 Hz, 1H, H-12a), 3.69 (dd, *J* = 17.7, 2.7 Hz, 1H, H-12b), 4.64 (dd, *J* = 12.3, 2.1 Hz, 1H, H-5b), 5.39 (d, *J* = 8.4 Hz, 1H, H-8), 5.49 (d, *J* = 8.1 Hz, 1H, H-7), 6.03–6.09 (m, 1H, ArH), 6.55 (s, 1H, H-25), 6.91–7.14 (m, 3H, ArH), 7.20–7.63 (m, 8H, ArH), 7.70 (d, *J* = 8.1 Hz, 1H, ArH), 7.81 (d, *J* = 7.8 Hz, 1H, ArH); ^13^C-NMR (75 MHz, CDCl_3_): δc 33.6, 52.1, 53.5, 53.7, 57.6, 75.5, 79.8, 95.2, 103.6, 121.6, 124.0, 124.2, 126.5, 126.9, 127.1, 127.5, 127.8, 128.2, 128.6, 128.8, 129.5, 129.8, 130.2, 131.7, 133.1, 133.6, 134.3, 134.5, 135.1, 135.8, 136.4, 137.3, 138.6, 194.3. Anal. calcd for C_34_H_26_Cl_2_N_2_O_2_S: C, 68.34; H, 4.39; N, 4.69. Found: C, 68.47; H, 4.30; N, 4.81%.

*8-(2-Fluorophenyl)-11-[(E)-(2-fluorophenyl)methylidene]-14-hydroxy-6-thia-3,13-diazaheptacyclo[13.7.1.1^9,13^.0^2,9^.0^2,14^.0^3,7^.0^19,23^]tetracosa-1(22),15(23),16,18,20-pentaen-10-one* (**6f**) Pale yellow solid, 90% (0.163 g), m.p. 162–164 °C, IR (KBr) υ_max_ 3418, 1724, 1690, 1599 cm^−1^; ^1^H-NMR (300 MHz, CDCl3): δ_H_ 2.77–3.01 (m, 4H, H-4a, H-4b, H-24a and H-24b), 3.13 (d, *J* = 12.6 Hz, 1H, H-5a), 3.24 (d, *J* = 17.7 Hz, 1H, H-12a), 3.65 (dd, *J* = 17.7, 2.1 Hz, 1H, H-12b), 4.53 (d, *J* = 12.6 Hz, 1H, H-5b), 4.98 (d, *J* = 7.2 Hz, 1H, H-8), 5.78 (d, *J* = 7.2 Hz, 1H, H-7), 5.96 (brs, 1H, OH), 6.17–6.24 (m, 2H, ArH and H-25), 6.77–6.86 (m, 2H, ArH), 7.08–7.32 (m, 5H, ArH), 7.54–7.64 (m, 5H, ArH), 7.80 (d, *J* = 7.8 Hz, 1H, ArH); ^13^C-NMR (75 MHz, CDCl_3_): δ_C_ 33.7, 50.4, 53.1, 53.4, 57.5, 57.6, 75.8, 94.7, 103.7, 115.5, 116.8, 121.6, 122.0, 123.6, 123.9, 124.2, 124.7, 126.3, 127.0, 128.0, 128.5, 129.7, 130.4, 130.5, 130.9, 131.2, 132.5, 135.2, 135.7, 137.3, 138.2, 160.2, 161.7, 195.7. Anal. calcd for C_34_H_26_F_2_N_2_O_2_S: C, 72.32; H, 4.64; N, 4.96. Found: C, 72.47; H, 4.52; N, 4.87%.

*14-Hydroxy-8-(3-nitrophenyl)-11-[(E)-(3-nitrophenyl)methylidene]-6-thia-3,13-diazaheptacyclo[13.7.1.1^9,13^.0^2,9^.0^2,14^.0^3,7^.0^19,23^]tetracosa-1(22),15(23),16,18,20-pentaen-10-one* (**6g**) Dark brown solid, 89% (0.150 g), m.p. 180–182 °C, IR (KBr) υ_max_ 3416, 1719, 1694, 1601 cm^−1^; ^1^H-NMR (400 MHz, CDCl_3_): δ_H_ 2.76–3.02 (m, 5H, H-4a, H-4b, H-5a, H-24a and H-24b), 3.31 (d, *J* = 17.6 Hz, 1H, H-12a), 3.67 (d, *J* = 17.6 Hz, 1H, H-12b), 4.36 (d, *J* = 12.0 Hz, 1H, H-5b), 4.97 (d, *J* = 7.2 Hz, 1H, H-8), 5.74 (d, *J* = 7.2 Hz , 1H, H-7), 6.15 (s, 1H, H-25), 6.71 (d, *J* = 7.2 Hz, 1H, ArH), 7.12 (s, 1H, ArH), 7.23–7.34 (m, 2H, ArH), 7.51–7.73 (m, 4H, ArH), 7.88 (d, *J* = 8.4 Hz, 1H, ArH), 7.93 (d, *J* = 8.0 Hz, 1H, ArH), 8.00 (d, *J* = 8.0 Hz, 1H, ArH), 8.17–8.24 (m, 2H, ArH), 8.46 (s, 1H, ArH); ^13^C-NMR (100 MHz, CDCl_3_): δ_C_ 34.0, 48.2, 52.9, 53.3, 53.6, 57.6, 75.5, 95.0, 103.9, 121.8, 123.0, 123.3, 123.8, 124.1, 124.5, 124.9, 126.2, 127.0, 128.1, 128.8, 129.3, 130.1, 131.2, 133.2, 133.8, 134.9, 135.6, 136.2, 136.5, 138.0, 139.4, 147.9, 148.7, 196.1. Anal. calcd for C_34_H_26_N_4_O_6_S: C, 66.01; H, 4.24; N, 9.06. Found: C, 66.15; H, 4.10; N, 9.21%.

*8-(2,4-Dichlorophenyl)-11-[(E)-(2,4-dichlorophenyl)methylidene]-14-hydroxy-6-thia-3,13-diazaheptacyclo[13.7.1.1^9,13^.0^2,9^.0^2,14^.0^3,7^.0^19,23^]tetracosa-1(22),15(23),16,18,20-pentaen-10-one* (**6h**) Light brown solid, 91% (0.147 g), m.p. 146–147 °C, IR (KBr) υ_max_ 3380, 1723, 1690, 1601 cm^−1^; ^1^H-NMR (400 MHz, CDCl_3_): δ_H_ 2.86–2.99 (m, 4H, H-4a, H-4b, H-24a, and H-24b), 3.21 (d, *J* = 12.0 Hz, 1H, H-5a), 3.33 (d, *J* = 18.0 Hz, 1H, H-12a), 3.66 (dd, *J* = 17.6, 2.8 Hz, 1H, H-12b), 4.59 (d, *J* = 12.4 Hz, 1H, H-5b), 5.30 (d, *J* = 8.4 Hz, 1H, H-8), 5.41 (d, *J* = 8.0 Hz 1H, H-7), 5.99 (d, *J* = 8.4 Hz 1H, ArH), 6.43 (s, 1H, H-25), 6.94 (dd, *J* = 8.4, 2.0 Hz, 1H, ArH), 7.15–7.28 (m, 2H, ArH), 7.37 (d, *J* = 8.8 Hz, 1H, ArH), 7.45 (d, *J* = 7.2 Hz, 1H, ArH), 7.51–7.62 (m, 4H, ArH), 7.71 (d, *J* = 8.4 Hz, 1H, ArH), 7.80 (dd, *J* = 6.4, 4.0 Hz, 1H, ArH); ^13^C-NMR (100 MHz, CDCl_3_): δ_C_ 33.5, 50.4, 52.7, 53.1, 57.4, 75.6, 78.7, 95.0, 103.5, 121.7, 124.1, 126.6, 126.7, 127.4, 127.5, 128.4, 128.7, 129.6, 129.8, 130.4, 130.9, 131.0, 131.3, 132.0, 133.6, 134.3, 134.6, 135.1, 135.2, 135.3, 136.9, 137.0, 138.1, 194.8. Anal. calcd for C_34_H_24_Cl_4_N_2_O_2_S: C, 61.28; H, 3.63; N, 4.20. Found: C, 61.15; H, 3.72; N, 4.35%.

*14-hydroxy-8-(4-methylphenyl)-11-[(E)-(4-methylphenyl)methylidene]-6-thia-3,13-diazaheptacyclo[13.7.1.1^9,13^.0^2,9^.0^2,14^.0^3,7^.0^19,23^]tetracosa-1(22),15(23),16,18,20-pentaen-10-one* (**6i**) Orange solid, 92% (0.168 g), m.p. 190–192 °C, IR (KBr) υ_max_ 3394, 1723, 1682, 1598 cm^−1^; ^1^H-NMR (300 MHz, CDCl_3_): δ_H_ 2.23 (s, 3H, CH_3_), 2.32 (s, 3H, CH_3_), 2.79–2.97 (m, 5H, H-4a, H-4b, H-5a, H-24a and H-24b), 3.40 (d, *J* = 17.4 Hz, 1H, H-12a), 3.67 (dd, *J* = 17.4, 2.8 Hz, 1H, H-12b), 4.23 (d, *J* = 12.3 Hz, 1H, H-5b), 4.87 (d, *J* = 7.2 Hz, 1H, H-8), 5.67 (d, *J* = 7.2 Hz, 1H, H-7), 6.29 (s, 1H, H-25), 6.36 (d, *J* = 8.1 Hz, 2H, ArH), 6.89 (d, *J* = 7.8 Hz, 2H, ArH), 7.15 (d, *J* = 8.1 Hz, 2H, ArH), 7.28–7.33 (m, 1H, ArH), 7.40 (d, *J* = 8.1 Hz, 2H, ArH), 7.52–7.61 (m, 4H, ArH), 7.72 (dd, *J* = 6.9, 1.8 Hz, 1H, ArH); ^13^C-NMR (75 MHz, CDCl_3_): δ_C_ 21.4, 21.7, 33.8, 53.1, 53.5, 53.7, 57.5, 75.0, 78.0, 95.0, 103.8, 121.6, 124.2, 126.3, 126.6, 128.1, 128.4, 128.9, 129.0, 129.7, 130.2, 131.2, 131.5, 132.3, 134.2, 136.1, 136.7, 137.2, 138.4, 139.2, 196.3. Anal. calcd for C_36_H_32_N_2_O_2_S: C, 77.67; H, 5.79; N, 5.03. Found: C, 77.84; H, 5.62; N, 5.16%.

*14-Hydroxy-8-(4-methoxyphenyl)-11-[(E)-(4-methoxyphenyl)methylidene]-6-thia-3,13-diazaheptacyclo[13.7.1.1^9,13^.0^2,9^.0^2,14^.0^3,7^.0^19,23^]tetracosa-1(22),15(23),16,18,20-pentaen-10-one* (**6j**) White solid, 86% (0.150 g), m.p. 144–146 °C, IR (KBr) υ_max_ 3398, 1719, 1681, 1600 cm^−1^; ^1^H-NMR (300 MHz, CDCl_3_): δ_H_ 2.80–2.99 (m, 5H, H-4a, H-4b, H-5a, H-24a and H-24b), 3.39 (d, *J* = 17.4 Hz, 1H, H-12a), 3.69 (dd, *J* = 17.4, 2.4 Hz, 1H, H-12b), 3.74 (s, 3H, OCH_3_), 3.79 (s, 3H, OCH_3_), 4.22 (d, *J* = 12.6, 2.1 Hz, 1H, H-5b), 4.85 (d, *J* = 7.5 Hz, 1H, H-8), 5.64 (d, *J* = 7.5 Hz, 1H, H-7), 5.95 (s, 1H, OH), 6.28 (s, 1H, H-25), 6.46 (d, *J* = 8.7 Hz, 2H, ArH), 6.62 (d, *J* = 8.7 Hz, 2H, ArH), 6.88 (d, *J* = 8.7 Hz, 2H, ArH), 7.27–7.35 (m, 1H, ArH), 7.43 (d, *J* = 8.7 Hz, 2H, ArH), 7.51–7.58 (m, 3H, ArH), 7.61 (d, *J* = 6.6 Hz, 1H, ArH), 7.71 (dd, *J* = 6.3, 2.7 Hz, 1H, ArH); ^13^C-NMR (75 MHz, CDCl_3_): δ_C_ 33.8, 53.1, 53.4, 53.5, 55.6, 55.7, 57.4, 74.9, 78.0, 95.0, 103.8, 113.8, 114.4, 121.6, 124.1, 126.3, 126.6, 127.0, 128.1, 128.4, 129.3, 130.0, 131.1, 131.2, 132.1, 136.1, 136.4, 137.2, 138.3, 159.2, 160.3, 196.3. Anal. calcd for C_36_H_32_N_2_O_4_S: C, 73.45; H, 5.48; N, 4.76. Found: C, 73.59; H, 5.32; N, 4.60%.

*8-(4-Bromophenyl)-11-[(E)-(4-bromophenyl)methylidene]-14-hydroxy-6-thia-3,13-diazaheptacyclo[13.7.1.1^9,13^.0^2,9^.0^2,14^.0^3,7^.0^19,23^]tetracosa-1(22),15(23),16,18,20-pentaen-10-one* (**6k**) Brown solid, 90% (0.142 g ), m.p. 156–158 °C, IR (KBr) υ_max_ 3390, 1723, 1681, 1603 cm^−1^; ^1^H-NMR (300 MHz, CDCl_3_): δ_H_ 2.83–2.95 (m, 5H, H-4a, H-4b, H-5a, H-24a and H-24b), 3.34 (d, *J* = 17.4 Hz, 1H, H-12a), 3.62 (d, *J* = 17.4, 2.1 Hz, 1H, H-12b), 4.20 (d, *J* = 12.6 Hz, 1H, H-5b), 4.83 (d, *J* = 7.2 Hz, 1H, H-8), 5.63 (d, *J* = 7.2 Hz, 1H, H-7), 6.17 (s, 1H, H-25), 6.27 (d, *J* = 8.4 Hz, 2H, ArH), 7.23 (d, *J* = 8.4 Hz, 2H, ArH), 7.32–7.41 (m, 3H, ArH), 7.49 (d, *J* = 8.4 Hz, 2H, ArH), 7.56–7.61 (m, 4H, ArH), 7.72–7.76 (m, 1H, ArH); ^13^C-NMR (75 MHz, CDCl_3_): δ_C_ 33.9, 53.1, 53.4, 53.5, 57.5, 75.1, 77.6, 95.0, 103.8, 121.8, 121.9, 123.3, 124.2, 126.4, 126.8, 128.3, 128.5, 130.7, 131.2, 131.4, 131.5, 132.2, 132.9, 133.5, 135.1, 135.8, 136.2, 137.1, 138.1, 196.1. Anal. calcd for C_34_H_26_Br_2_N_2_O_2_S: C, 59.49; H, 3.82; N, 4.08. Found: C, 59.40; H, 3.71; N, 4.24%.

*8-(4-Chlorophenyl)-11-[(E)-(4-chlorophenyl)methylidene]-14-hydroxy-6-thia-3,13-diazaheptacyclo[13.7.1.1^9,13^.0^2,9^.0^2,14^.0^3,7^.0^19,23^]tetracosa-1(22),15(23),16,18,20-pentaen-10-one* (**6l**) Brown solid, 92% (0.160 g ), m.p. 164–166 °C, IR (KBr) υ_max_ 3357, 1719, 1682, 1605 cm^−1^; ^1^H-NMR (400 MHz, CDCl_3_): δ_H_ 2.84–2.94 (m, 5H, H-4a, H-4b, H-5a, H-24a and H-24b), 3.35 (d, *J* = 17.6 Hz, 1H, H-12a), 3.63 (d, *J* = 17.6, 2.4 Hz, 1H, H-12b), 4.28 (d, *J* = 12.4 Hz, 1H, H-5b), 4.83 (d, *J* = 6.4 Hz, 1H, H-8), 5.65 (d, *J* = 6.4 Hz, 1H, H-7), 6.02 (s, 1H, OH), 6.15 (s, 1H, H-25), 6.71 (d, *J* = 7.6 Hz, 1H, ArH), 7.12 (s, 1H, ArH), 7.23–7.34 (m, 2H, ArH), 7.51–7.73 (m, 4H, ArH), 7.88 (d, *J* = 8.4 Hz, 2H, ArH), 7.93 (d, *J* = 8.0 Hz, 1H, ArH), 8.00 (d, *J* = 8.0 Hz, 1H, ArH), 8.17–8.24 (m, 2H, ArH ); ^13^C-NMR (100 MHz, CDCl_3_): δ_C_ 33.8, 52.7, 53.0, 53.3, 57.3, 75.2, 78.2, 94.7, 103.9, 121.4, 123.8, 126.1, 126.5, 127.9, 128.2, 128.3, 128.8, 130.3, 130.9, 131.0, 132.3, 133.2, 133.9, 134.4, 134.5, 135.9, 136.0, 136.9, 138.2, 196.1. Anal. calcd for C_34_H_26_Cl_2_N_2_O_2_S: C, 68.34; H, 4.39; N, 4.69. Found: C, 68.20; H, 4.57; N, 4.60%.

*8-(4-Fluorophenyl)-11-[(E)-(4-fluorophenyl)methylidene]-14-hydroxy-6-thia-3,13-diazaheptacyclo[13.7.1.1^9,13^.0^2,9^.0^2,14^.0^3,7^.0^19,23^]tetracosa-1(22),15(23),16,18,20-pentaen-10-one* (**6m**) Light brown solid, 91% (0.165 g), m.p. 136–138 °C, IR (KBr) υ_max_ 3424, 1723, 1682, 1598 cm^−1^; ^1^H-NMR (400 MHz, CDCl_3_): δ_H_ 2.85–2.99 (m, 5H, H-4a, H-4b, H-5a, H-24a and H-24b), 3.35 (d, *J* = 17.6 Hz, 1H, H-12a), 3.65 (dd, *J* = 17.6, 2.4 Hz, 1H, H-12b), 4.23 (dd, *J* = 12.8, 2.4 Hz, 1H, H-5b), 4.86 (d, *J* = 7.2 Hz, 1H, H-8), 5.64 (d, *J* = 7.6 Hz, 1H, H-7), 6.20 (s, 1H, H-25), 6.39–6.43 (m, 2H, ArH), 6.77–6.82 (m, 2H, ArH), 7.03–7.08 (m, 2H, ArH), 7.27–7.34 (m, 1H, ArH), 7.48–7.62 (m, 6H, ArH), 7.75 (dd, *J* = 6.4, 2.4, 1H, ArH); ^13^C-NMR (100 MHz, CDCl_3_): δ_C_ 33.9, 52.6, 53.1, 53.4, 57.6, 75.1, 77.9, 95.0, 103.8, 115.4, 115.9, 121.7, 124.1, 126.3, 126.8, 128.1, 128.4, 130.2, 130.6, 131.2, 131.9, 132.0, 133.1, 135.3, 135.9, 137.2, 138.1, 162.5, 162.9, 196.3. Anal. calcd for C_34_H_26_F_2_N_2_O_2_S: C, 72.32; H, 4.64; N, 4.96. Found: C, 73.45; H, 4.73; N, 4.82%.

## 4. Conclusions

An efficient three-component domino protocol has been achieved for the stereoselective synthesis of novel heptacyclic cage-like compounds in ionic liquid under microwave irradiation conditions. The similar reactivity found for azomethine ylides generated from thiazolidine-2-carboxylic acid and thiazolidine-4-carboxylic acid allowed discarding any influence on dipolar cycloadditions of the well-known [36] carbanion stabilization by adjacent sulfur effect. Further studies on the synthetic applications of this methodology with diverse 1,2-diketones and α-amino acids are currently under progress in our laboratories.

## Supplementary Materials

Supplementary materials can be accessed at: http://www.mdpi.com/1420-3049/21/2/165/s1.

## References

[1] Snyder S.A., Breazzano S.P., Ross A.G., Lin Y., Zografos A.L. (2009). Total synthesis of diverse carbogenic complexity within the resveratrol class from a common building block. J. Am. Chem. Soc..

[2] Wender P.A., Gamber G.G., Hubbard R.D., Pham S.M., Zhang L. (2005). Multicomponent cycloadditions: The four-component [5+1+2+1] cycloaddition of vinylcyclopropanes, alkynes, and CO. J. Am. Chem. Soc..

[3] Trost B.M. (1995). Atom economy—A challenge for organic synthesis: Homogeneous catalysis leads the way. Angew. Chem. Int. Ed. Engl..

[4] Tietze L.F., Brazel C.C., Holsken S., Magull J., Ringe A. (2008). Total synthesis of polyoxygenated cembrenes. Angew. Chem. Int. Ed..

[5] Lu M., Zhu D., Lu Y., Hou B., Tan B., Zhong G. (2008). Organocatalytic asymmetric alpha-aminoxylation/aza-Michael reactions for the synthesis of functionalized tetrahydro-1,2-oxazines. Angew. Chem. Int. Ed..

[6] Tietze L.F. (1996). Domino reactions in organic synthesis. Chem. Rev..

[7] Schwier T., Sromek A.W., Chernyak D., Gevorgyan V. (2007). Mechanistically diverse copper-, silver-, and gold-catalyzed acyloxy and phosphatyloxy migrations: Efficient synthesis of heterocycles via cascade migration/cycloisomerization approach. J. Am. Chem. Soc..

[8] Waldmann H., Kuhn M., Liu W., Kumar K. (2008). Reagent-controlled domino synthesis of skeletally-diverse compound collections. Chem. Commun..

[9] Tietze L.F., Haunert F., Vögtle F., Stoddart J.F., Shibasaki M. (2000). Stimulating Concepts in Chemistry.

[10] Gao J., Song Q.-W., He L.-N., Liu C., Yang Z.-Z., Han X., Li X.-D., Song Q.-C. (2012). Preparation of polystyrene-supported Lewis acidic Fe(III) ionic liquid and its application in catalytic conversion of carbon dioxide. Tetrahedron.

[11] Narayana Kumar G.G.K.S., Aridoss G., Laali K.K. (2012). Condensation of propargylic alcohols with indoles and carbazole in [bmim][PF_6_]/Bi(NO_3_)_3_·5H_2_O: A simple high yielding propargylation method with recycling and reuse of the ionic liquid. Tetrahedron Lett..

[12] Isambert N., Duque M.M.S., Plaquevent J.C., Genisson Y., Rodriguez J., Constantieux T. (2011). Multicomponent reactions and ionic liquids: A perfect synergy for eco-compatible heterocyclic synthesis. Chem. Soc. Rev..

[13] Santagada V., Perissutti E., Caliendo G. (2002). The application of microwave irradiation as new convenient synthetic procedure in drug discovery. Curr. Med. Chem..

[14] Loupy A. (2002). Microwaves in Organic Synthesis.

[15] De la Hoz A., Díaz-Ortiz A., Moreno A., Langa F. (2000). Cycloadditions under microwave irradiation conditions: Methods and applications. Eur. J. Org. Chem..

[16] Geldenhuys W.J., Malan S.F., Bloomquist J.R., Marchand A.P., van der Schyf C.J. (2005). Pharmacology and structure-activity relationships of bioactive polycyclic cage compounds: A focus on pentacycloundecane derivatives. Med. Res. Rev..

[17] Han Q.B., Xu H.X. (2009). Caged Garcinia xanthones: Development since 1937. Curr. Med. Chem..

[18] Chi Y., Zhan X.K., Yu H., Xie G.R., Wang Z.Z., Xiao W., Wang Y.G., Xiong F.X., Hu J.F., Yang L. (2013). An open-labeled, randomized, multicenter phase IIa study of gambogic acid injection for advanced malignant tumors. Chin. Med. J. (Engl.).

[19] Wang J., Zhao L., Hu Y., Guo Q., Zhang L., Wang X., Li N., You Q. (2009). Studies on chemical structure modification and biology of a natural product, Gambogic acid (I): Synthesis and biological evaluation of oxidized analogues of gambogic acid. Eur. J. Med. Chem..

[20] Suresh Kumar R., Almansour A.I., Arumugam N., Ali M.A. (2014). An expedient synthesis and screening for antiacetylcholinesterase activity of piperidine-embedded novel pentacyclic cage compounds. Med. Chem..

[21] Suresh Kumar R., Ali M.A., Osman H., Ismail R., Choon T.S., Yoon Y.K., Wei A.C., Pandian S., Manogaran E. (2011). Synthesis and discovery of novel hexacyclic cage compounds as inhibitors of acetylcholinesterase. Bioorg. Med. Chem. Lett..

[22] Suresh Kumar R., Osman H., Perumal S., Menéndez J.C., Ali M.A., Ismail R., Choon T.S. (2011). A facile three-component [3+2]-cycloaddition/annulation domino protocol for the regio- and diastereoselective synthesis of novel penta- and hexacyclic cage systems, involving the generation of two heterocyclic rings and five contiguous stereocenters. Tetrahedron.

[23] Suresh Kumar R., Almansour A.I., Arumugam N., Menéndez J.C., Osman H., Ranjith Kumar R. (2015). Dipolar cycloaddition-based multicomponent reactions in ionic liquids: A green, fully stereoselective synthesis of novel polycyclic cage systems with the generation of two new azaheterocyclic rings. Synthesis.

[24] Suresh Kumar R., Almansour A.I., Arumugam N., Basiri A., Kia Y., Ranjith Kumar R. (2015). Ionic liquid-promoted synthesis and cholinesterase inhibitory activity of highly functionalized spiropyrrolidines. Aust. J. Chem..

[25] Almansour A.I., Suresh Kumar R., Arumugam N., Basiri A., Kia Y., Ali M.A., Farooq M., Murugaiyah V. (2015). A facile ionic liquid promoted synthesis, cholinesterase inhibitory activity and molecular modeling study of novel highly functionalized spiropyrrolidines. Molecules.

[26] Malathi K., Kanchithalaivan S., Ranjith Kumar R., Almansour A.I., Suresh Kumar R., Arumugam N. (2015). Multicomponent [3+2] cycloaddition strategy: Stereoselective synthesis of novel polycyclic cage-like systems and dispiro compounds. Tetrahedron Lett..

[27] Arumugam N., Almansour A.I., Suresh Kumar R., Menéndez J.C., Sultan M.A., Karama U., Ghabbour H.A., Fun H.-K. (2015). An expedient regio- and diastereoselective synthesis of hybrid frameworks with embedded spiro[9,10]dihydroanthracene [9,3′]-pyrrolidine and spiro[oxindole-3,2′-pyrrolidine] motifs via an ionic liquid-mediated multicomponent reaction. Molecules.

[28] Almansour A.I., Suresh Kumar R., Arumugam N., Basiri A., Kia Y., Ali M.A. (2015). An expedient synthesis, acetylcholinesterase inhibitory activity, and molecular modeling study of highly functionalized hexahydro-1,6-naphthyridines. BioMed. Res. Int..

[29] Almansour A.I., Suresh Kumar R., Beevi F., Shirazi A.N., Osman H., Ismail R., Choon T.S., Sullivan B., McCaffrey K., Nahhas A. (2014). Facile, regio- and diastereoselective synthesis of spiro-pyrrolidine and pyrrolizine derivatives and evaluation of their antiproliferative activities. Molecules.

[30] Kumar R.S., Ramar A., Perumal S., Almansour A.I., Arumugam N., Ali M.A. (2013). Three-component synthesis and 1,3-dipolar cycloaddition of highly functionalized pyrans with nitrile oxides: Easy access to 1,2,4-oxadiazoles. Synth. Commun..

[31] Arumugam N., Almansour A.I., Kumar R.S., Perumal S., Ghabbour H.A., Fun H.-K. (2013). A 1,3-dipolar cycloaddition–annulation protocol for the expedient regio-, stereo- and product-selective construction of novel hybrid heterocycles comprising seven rings and seven contiguous stereocentres. Tetrahedron Lett..

[32] Dimmock J.R., Padmanilayam M.P., Puthucode R.N., Nazarali A.J., Motaganahalli N.L., Zello G.A., Quail J.W., Oloo E.O., Kraatz H.B., Prisciak J.S. (2001). A conformational and structure-activity relationship study of cytotoxic 3,5-bis(arylidene)-4-piperidones and related *N*-acryloyl analogues. J. Med. Chem..

[33] Suresh Kumar R., Osman H., Abdul Rahim A.S., Hemamalini M., Fun H.-K. (2011). 14-Hydroxy-11-[(*E*)-4-methoxybenzylidene]-8-(4-methoxyphenyl)-5-thia-3,13-diazaheptacyclo-[13.7.1.1^9,13^.0^2,9^.0^2,14^.0^3,7^.0^19,23^]tetracosa-1(22),15(23),16,-18,20-pentaen-10-one. Acta Crystallogr..

[34] Suresh Kumar R., Osman H., Almansour A.I., Arshad S., Razak I.A. (2012). 11-[(*E*)-2-Fluorobenzylidene]-8-(2-fluorophenyl)-14-hydroxy-6-thia-3,13-diazaheptacyclo-[13.7.1.1^9,13^.0^2,9^.0^2,14^.0^3,7^.0^19,23^]tetracosa-1(22),15(23),16,18,20-pentaen-10-one. Acta Crystallogr..

[35] Haddad S., Boudriga S., Porzio F., Soldera A., Askri M., Knorr M., Rousselin Y., Kubicki M.M., Golz C., Strohmann C. (2015). Regio- and stereoselective synthesis of spiropyrrolizidines and piperazines through azomethine ylide cycloaddition reaction. J. Org. Chem..

[36] Bernasconi C.F., Kittredge K.W. (1998). Carbanion stabilization by adjacent sulfur: Polarizability, resonance, or negative hyperconjugation? Experimental distinction based on intrinsic rate constants of proton transfer from (phenylthio)nitromethane and 1-nitro-2-phenylethane. J. Org. Chem..

