# Supplementary material for: A Sustainable Approach to the Stereoselective Synthesis of Diazaheptacyclic Cage Systems Based on a Multicomponent Strategy in an Ionic Liquid
Source: Molecules. 2016 Jan 29;21(2):165. doi: 10.3390/molecules21020165 (PMC6273701; doi:10.3390/molecules21020165)
Supplement: Supplementary file 1 [file molecules-21-00165-s001.pdf]

Supplementary Materials: A Sustainable Approach to the Stereoselective Synthesis of Diazaheptacyclic Cage Systems Based on a Multicomponent Strategy in an Ionic Liquid

Raju Suresh Kumar, Abdulrahman I. Almansour, Natarajan Arumugam, Mohammad Altaf, José Carlos Menéndez, Raju Ranjith Kumar and Hasnah Osman

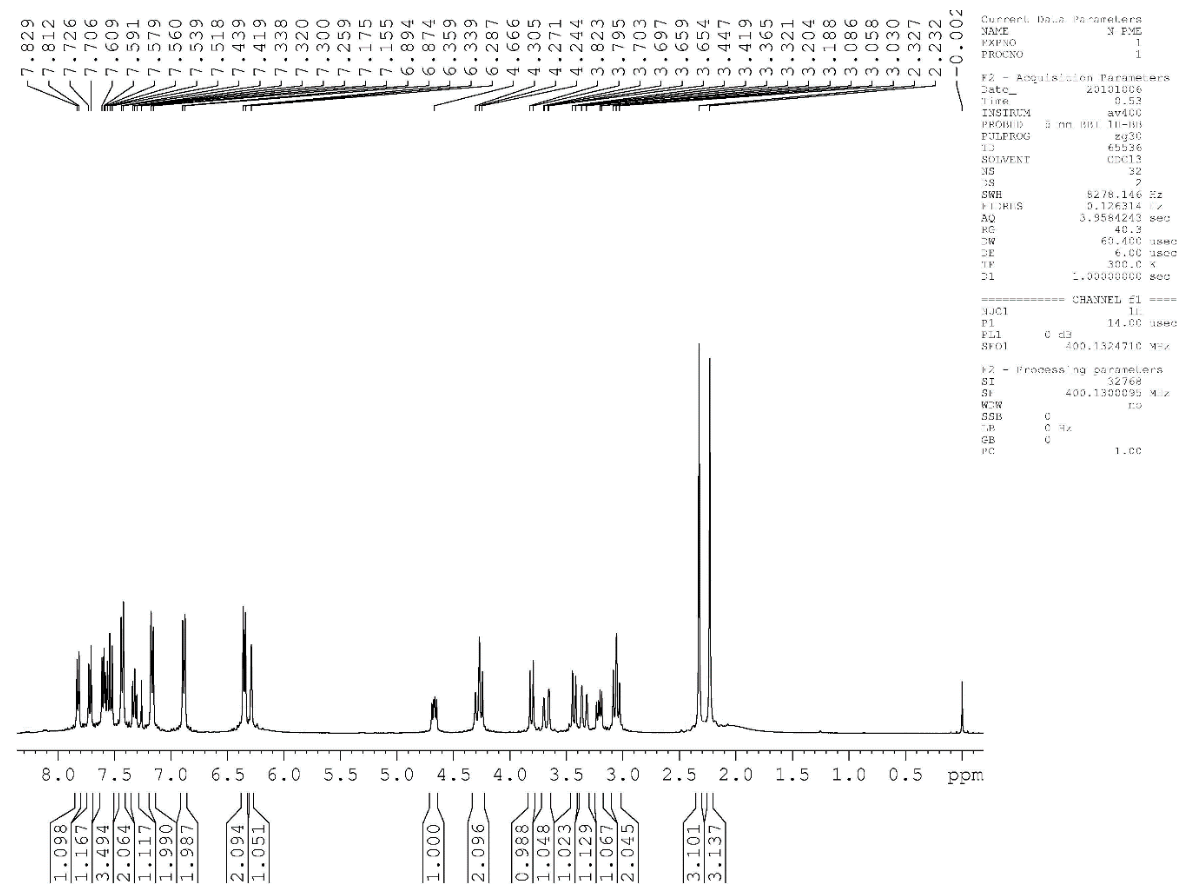

Figure 1. ^{1}H-NMR spectrum of **4i**.

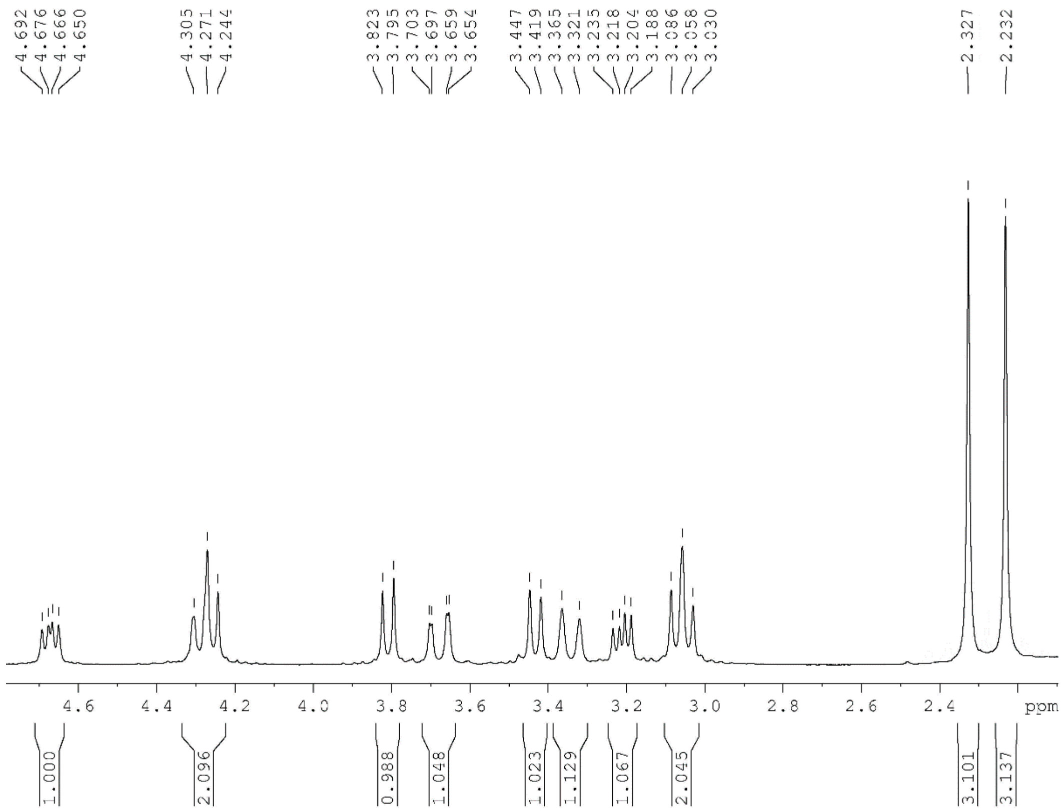

Figure 2. ^{1}H-NMR spectrum of **4i** (Expansion).

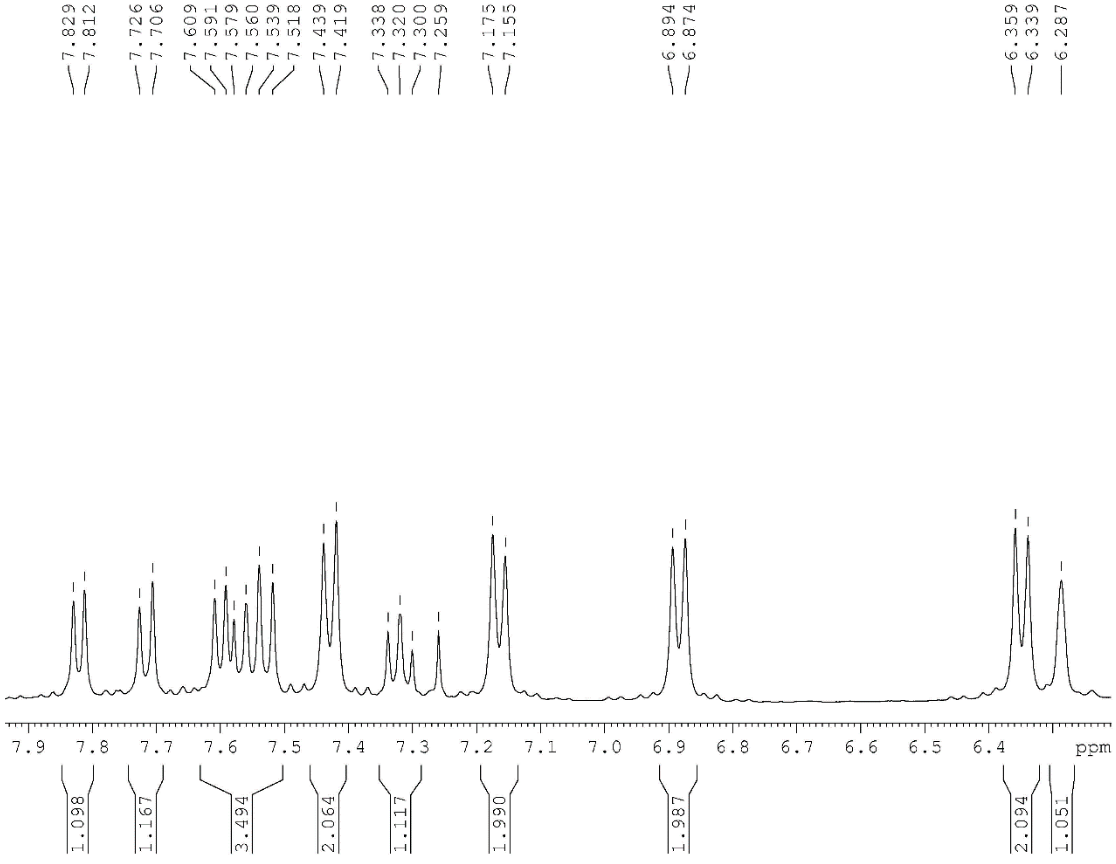

Figure 3. ^{1}H-NMR spectrum of **4i** (Expansion).

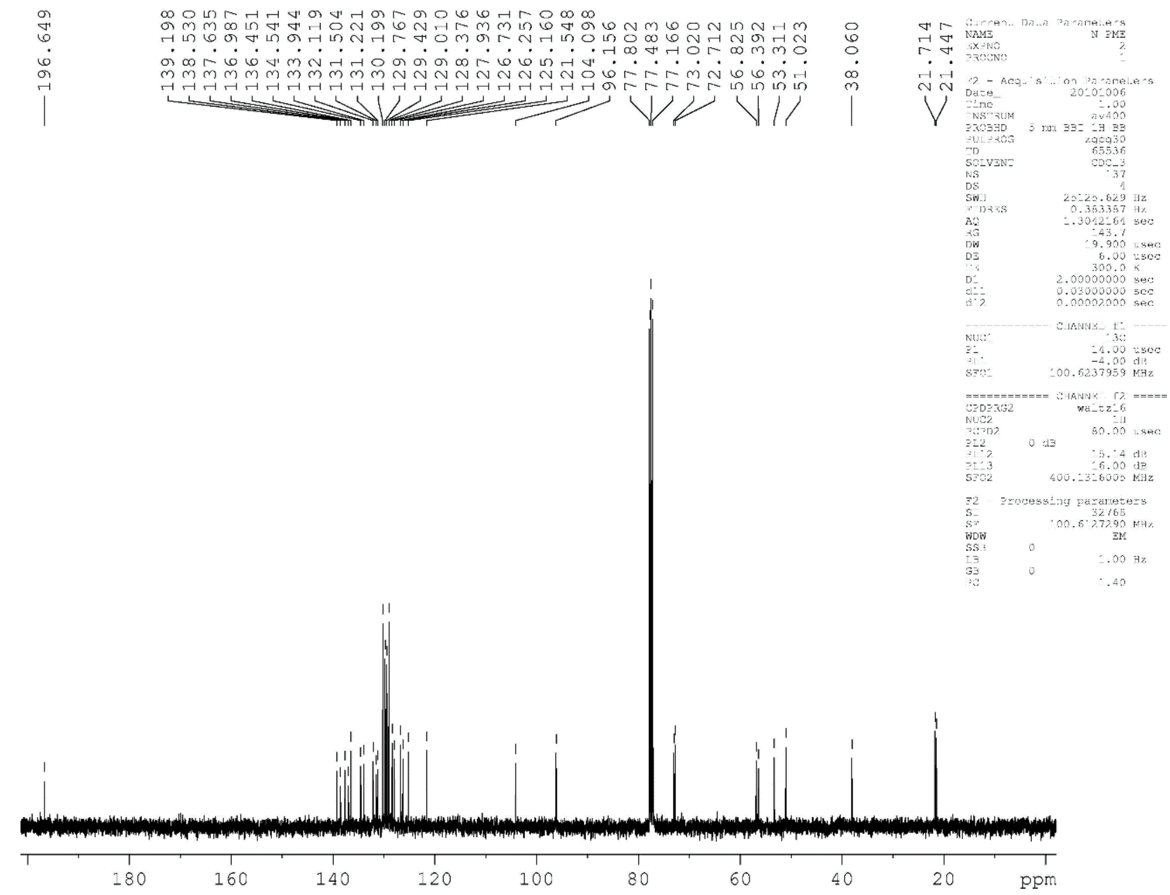

Figure 4. ^{13}C-NMR spectrum of **4i**.

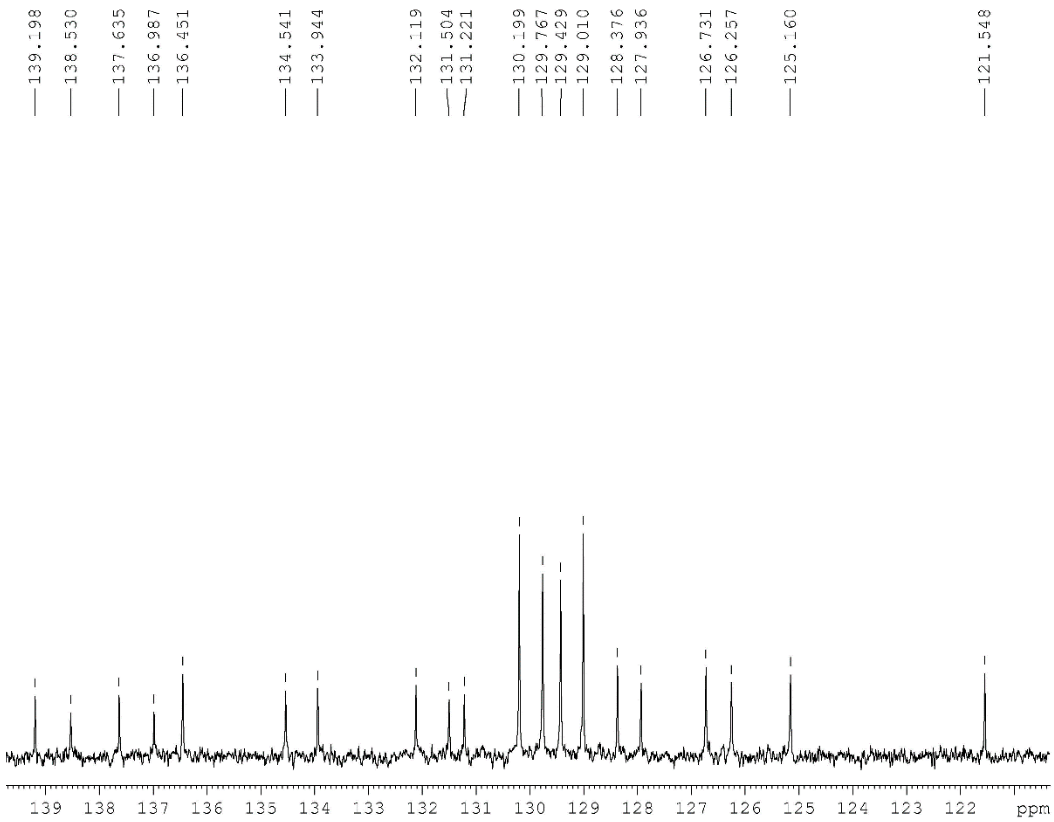

Figure 5. ^{13}C-NMR spectrum of **4i** (Expansion).

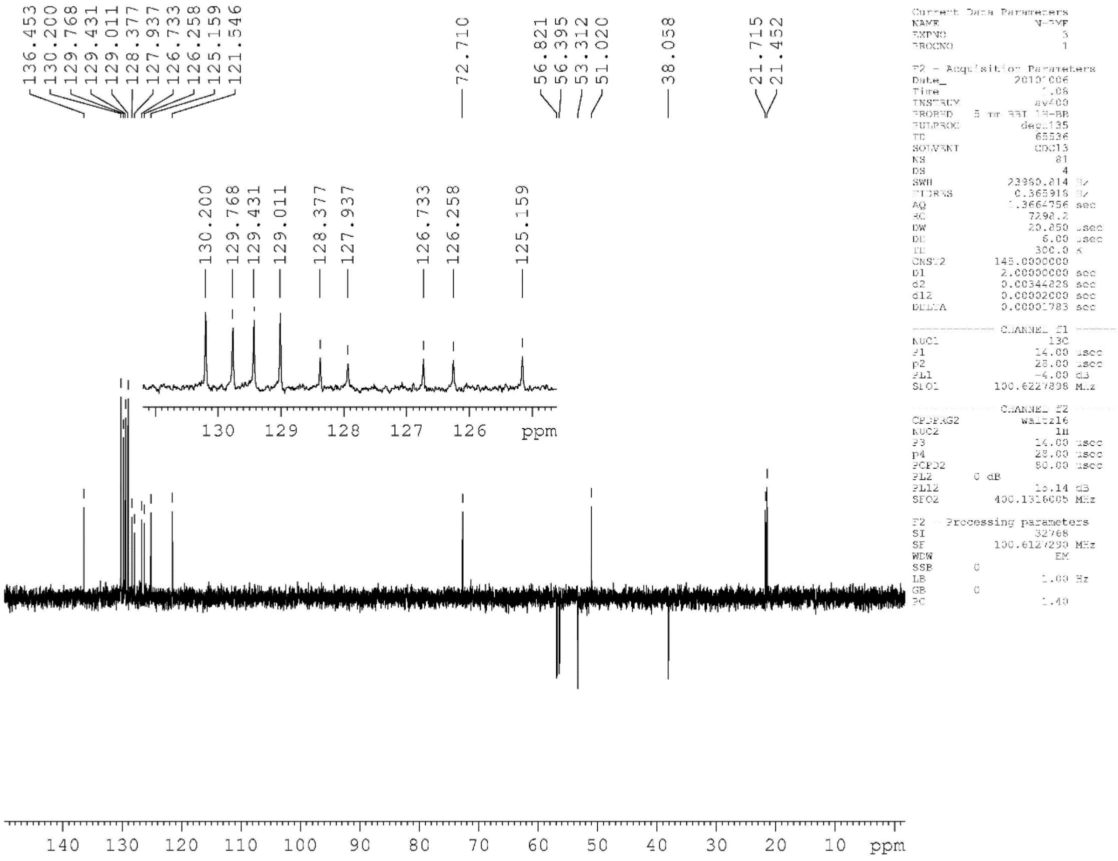

Figure 6. DEPT-135 spectrum of **4i**.

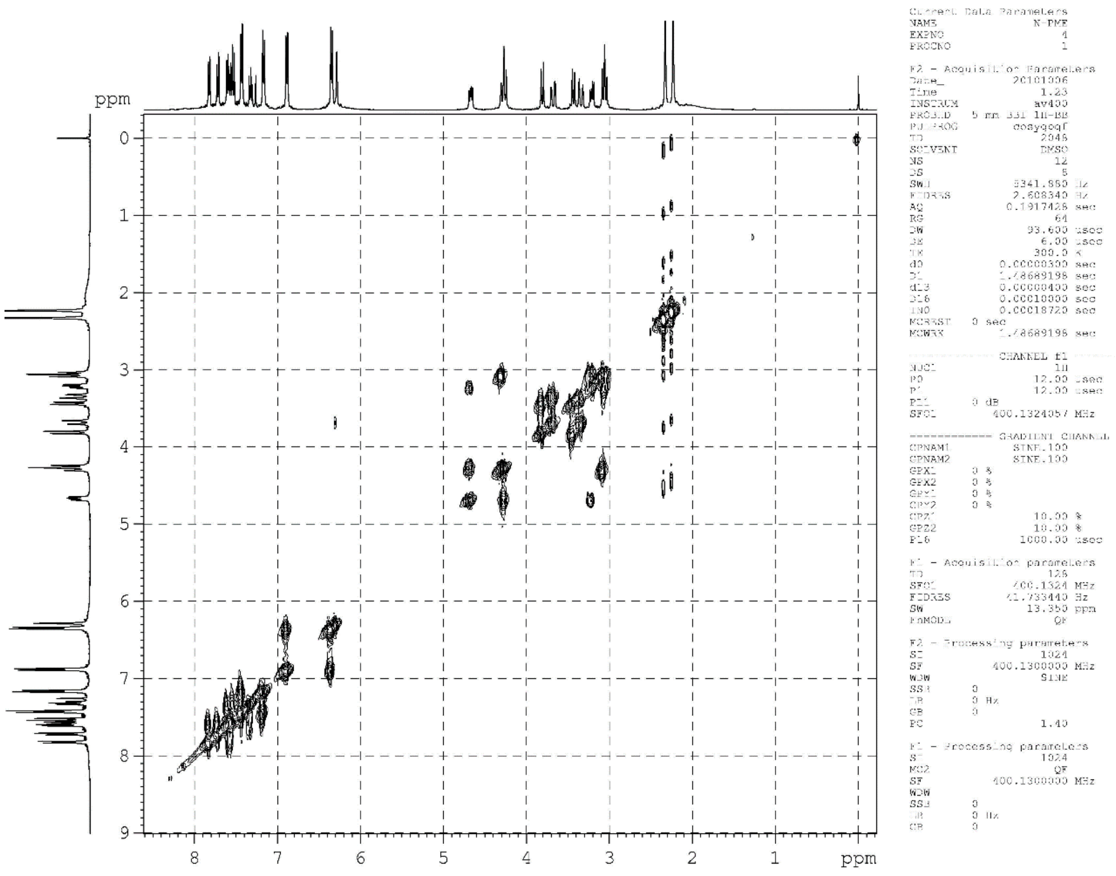

Figure 7. H,H-COSY spectrum of **4i**.

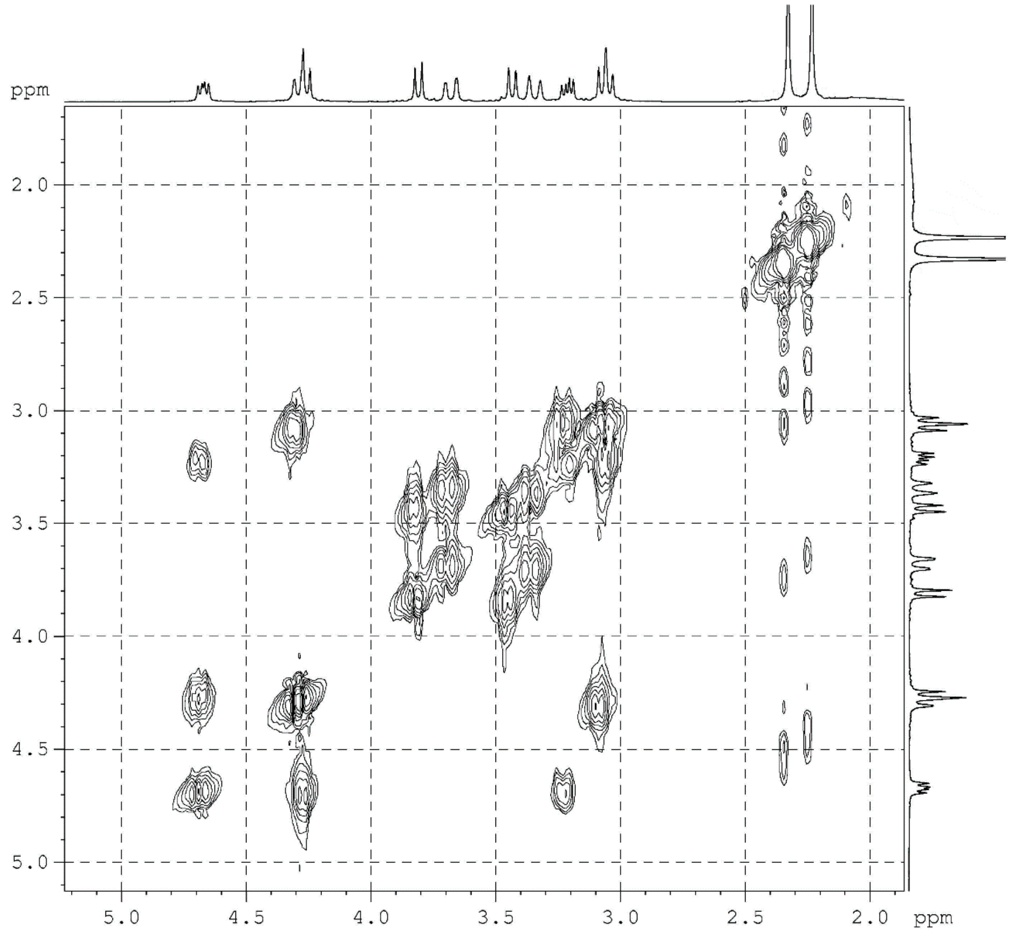

Figure 8. H,H-COSY spectrum of **4i** (Expansion).

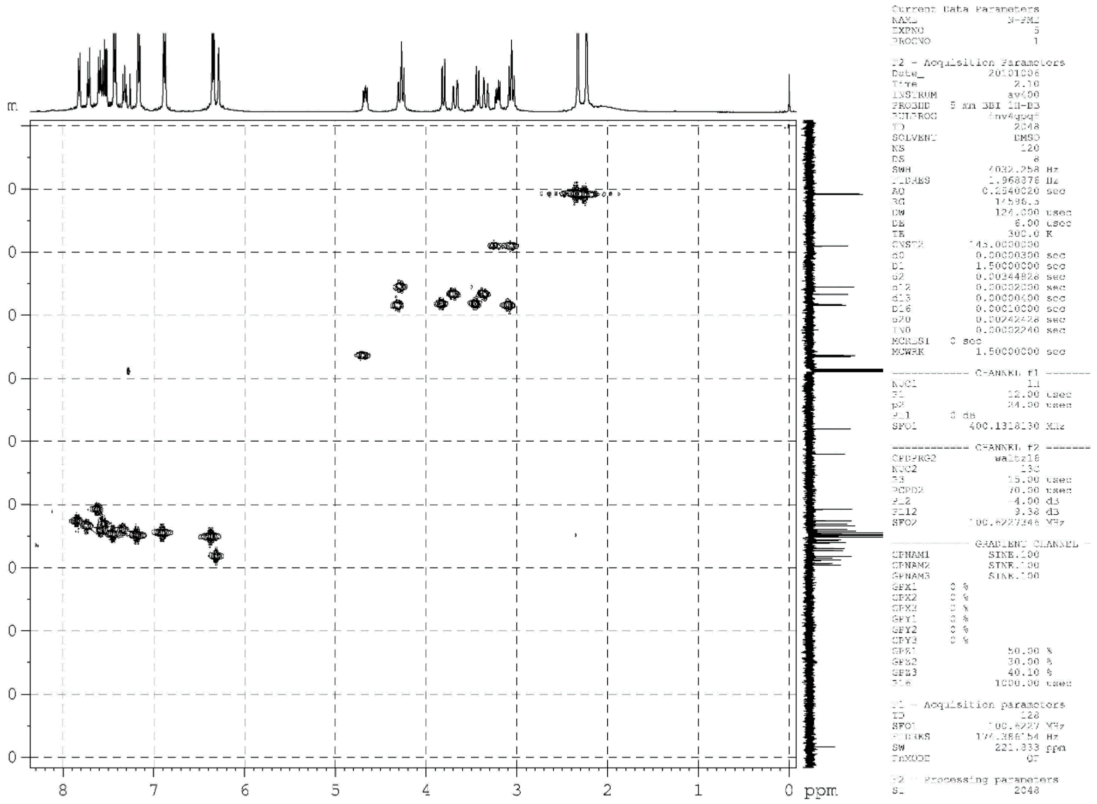

Figure 9. C,H-COSY spectrum of **4i**.

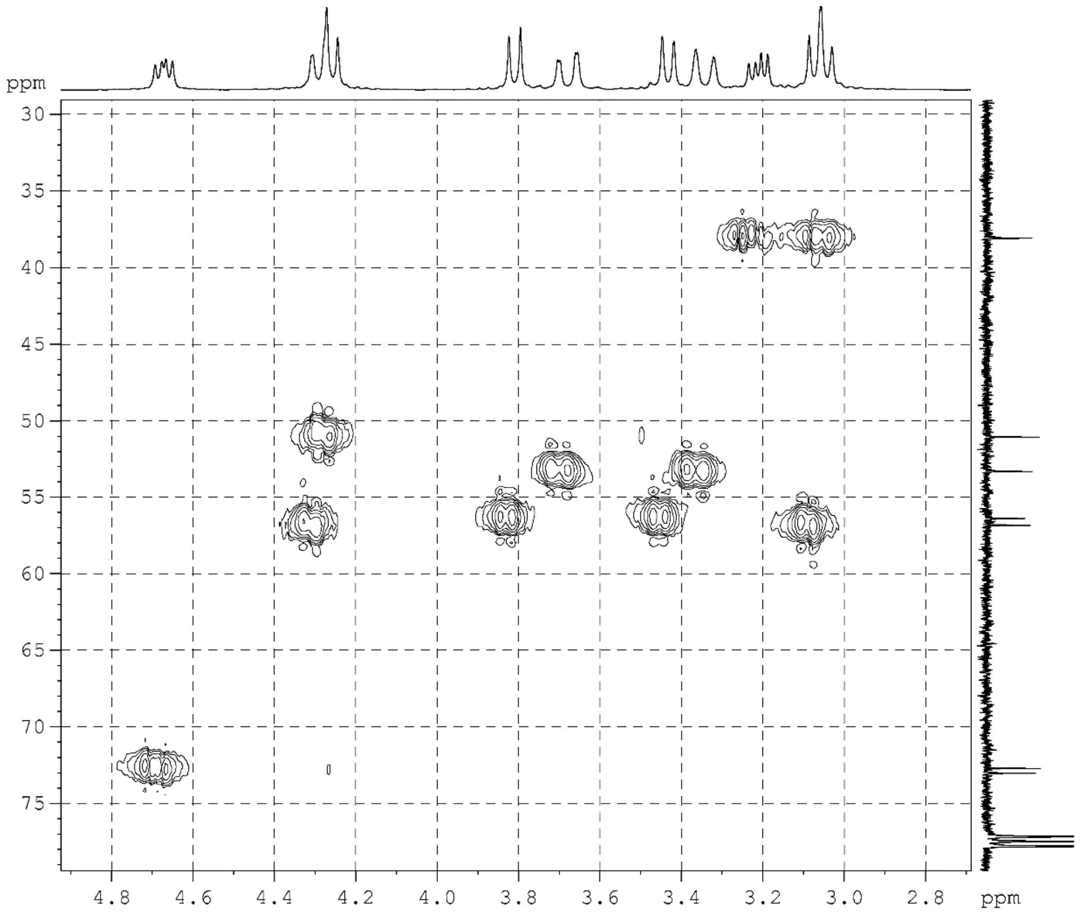

Figure 10. C,H-COSY spectrum of **4i** (Expansion).

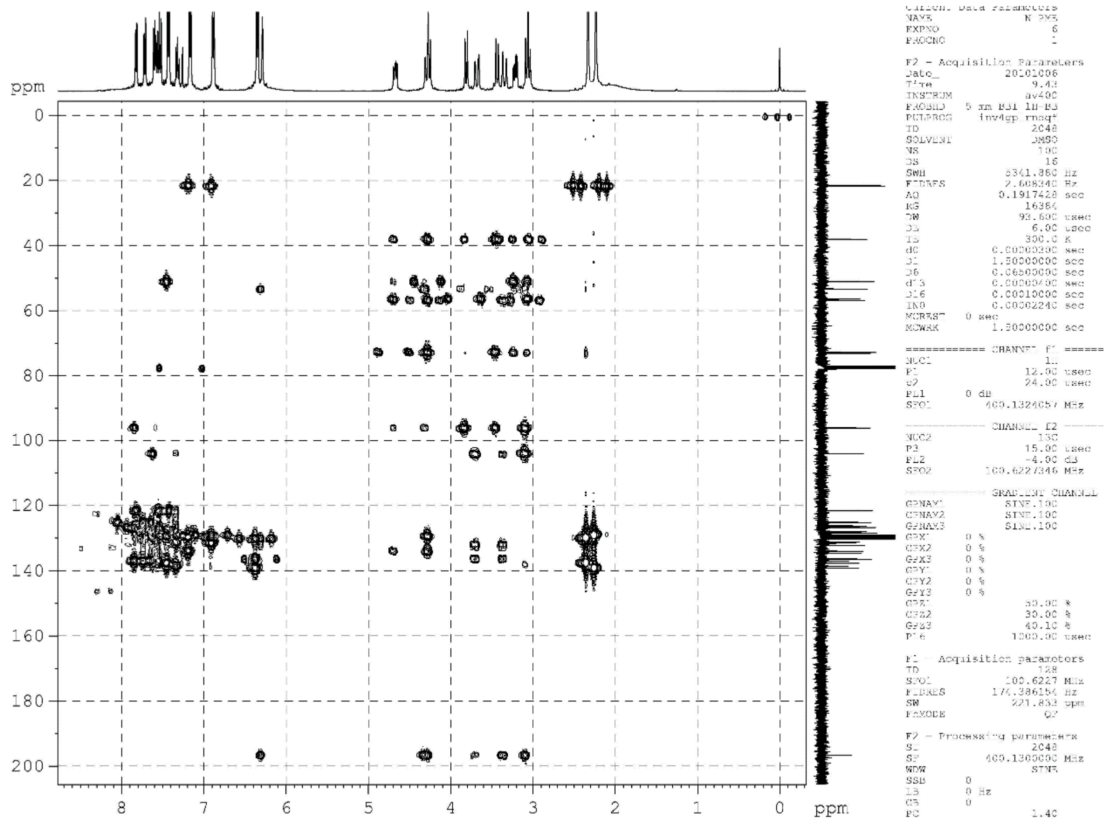

Figure 11. HMBC spectrum of **4i**.

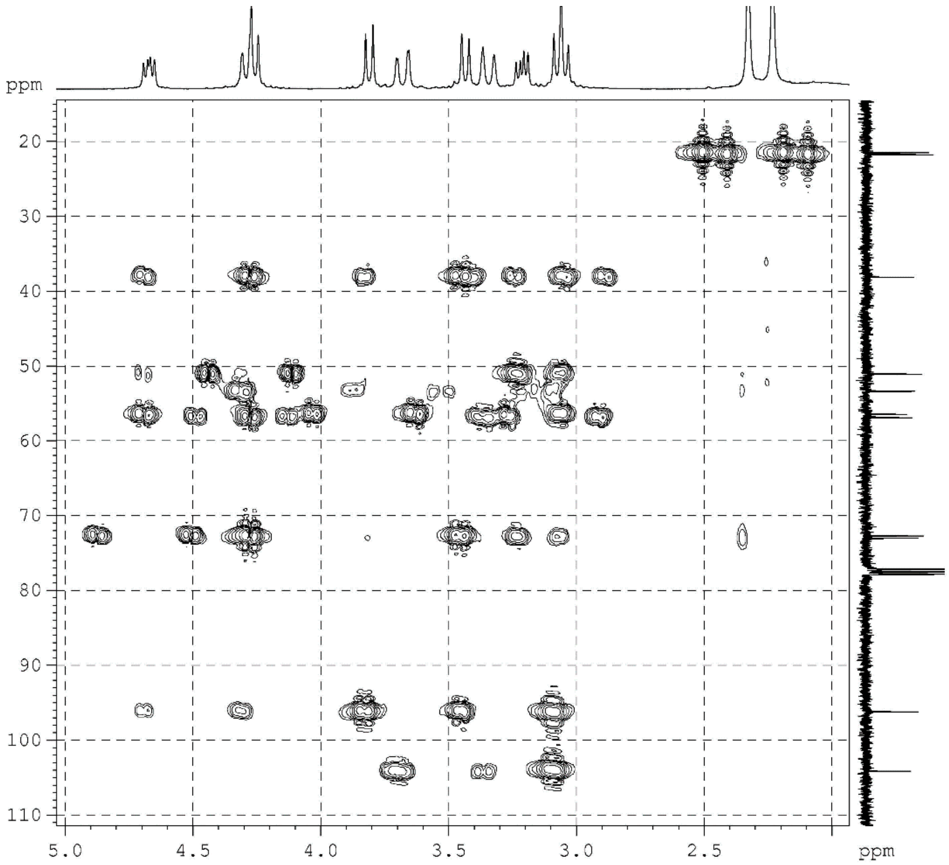

Figure 12. HMBC spectrum of **4i** (Expansion).

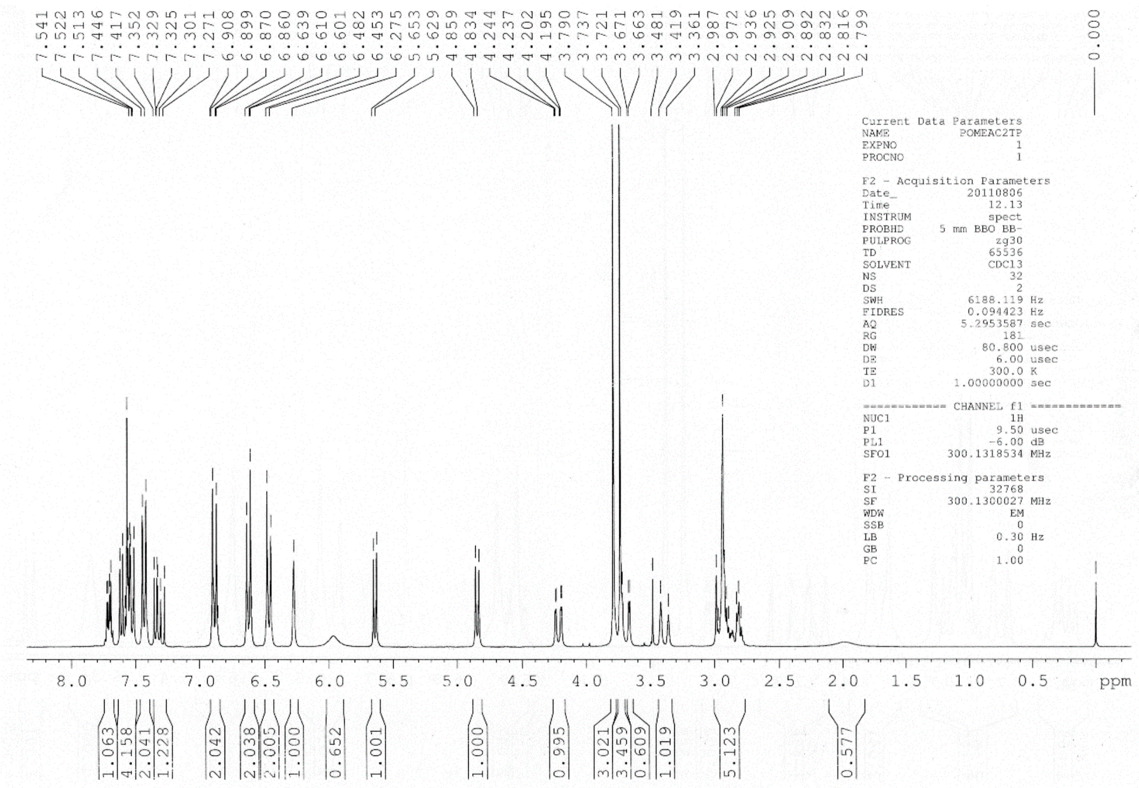

Figure 13. ^{1}H-NMR spectrum of **6j**.

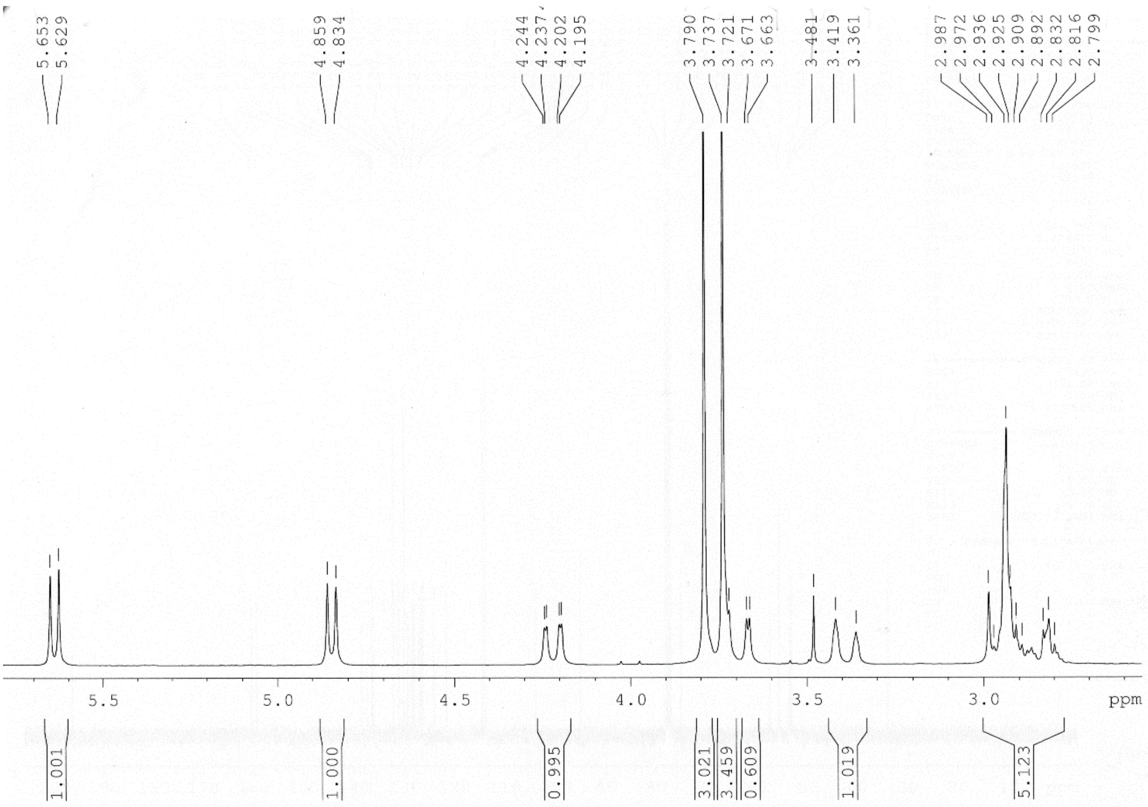

Figure 14. ^{1}H-NMR spectrum of **6j** (Expansion).

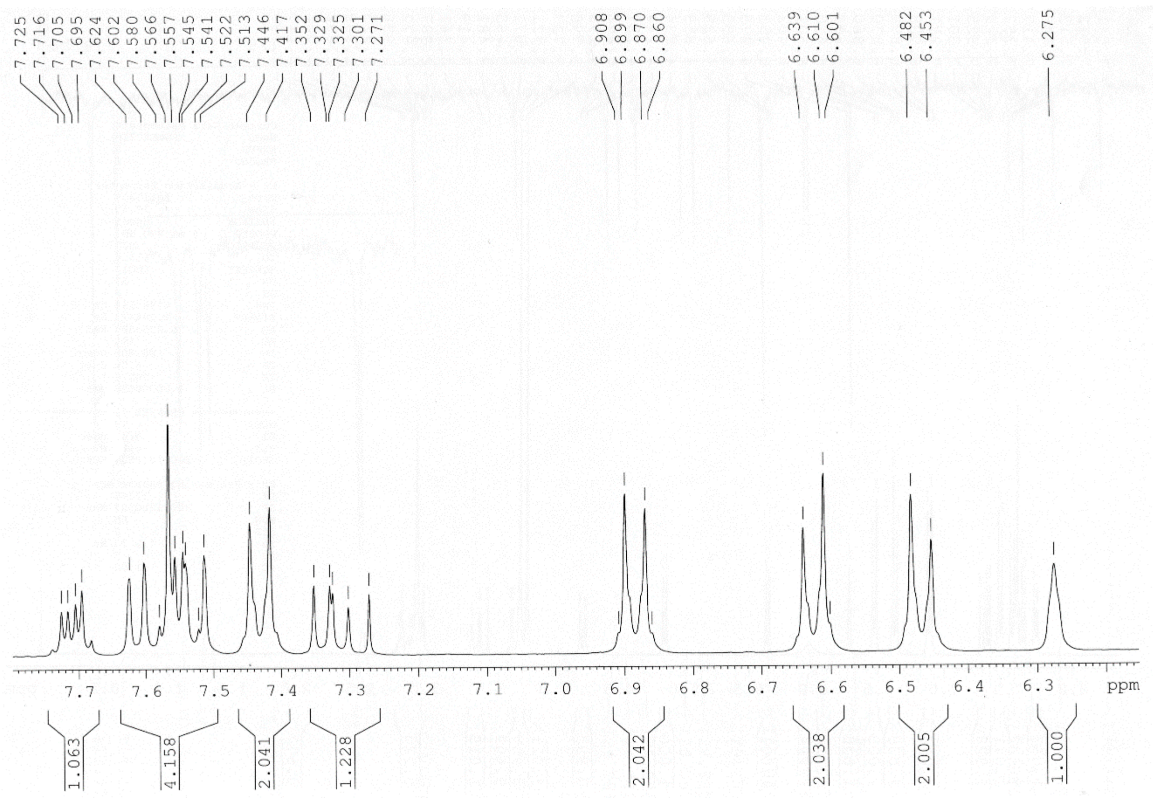

Figure 15. ^{1}H-NMR spectrum of **6j** (Expansion).

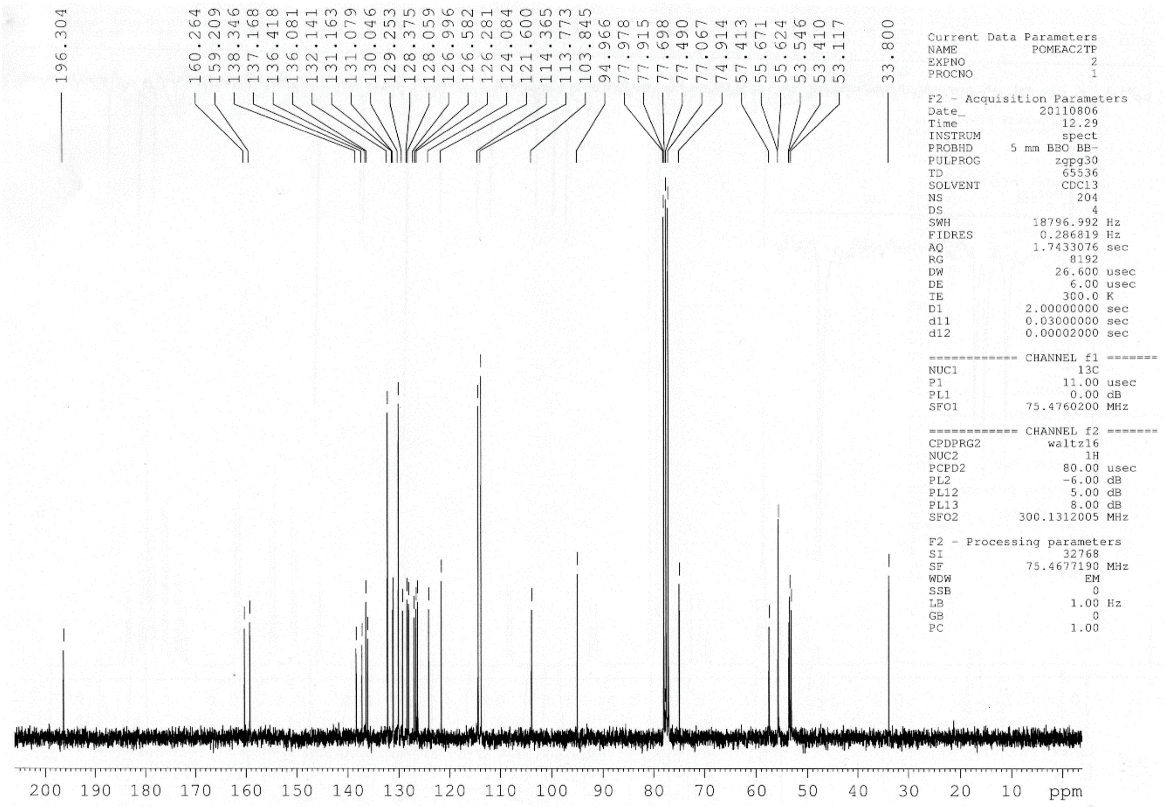

Figure 16. ^{13}C-NMR spectrum of **6j**.

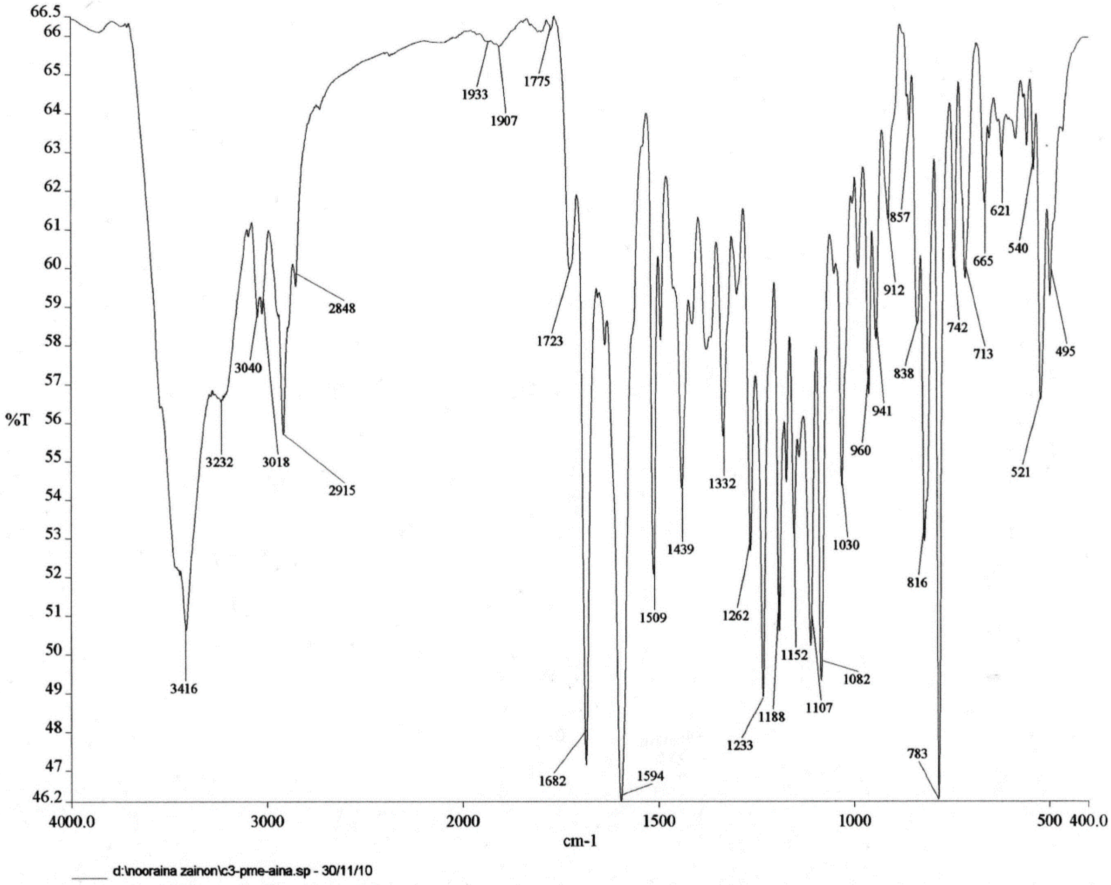

Figure 17. IR spectrum of **4i**.

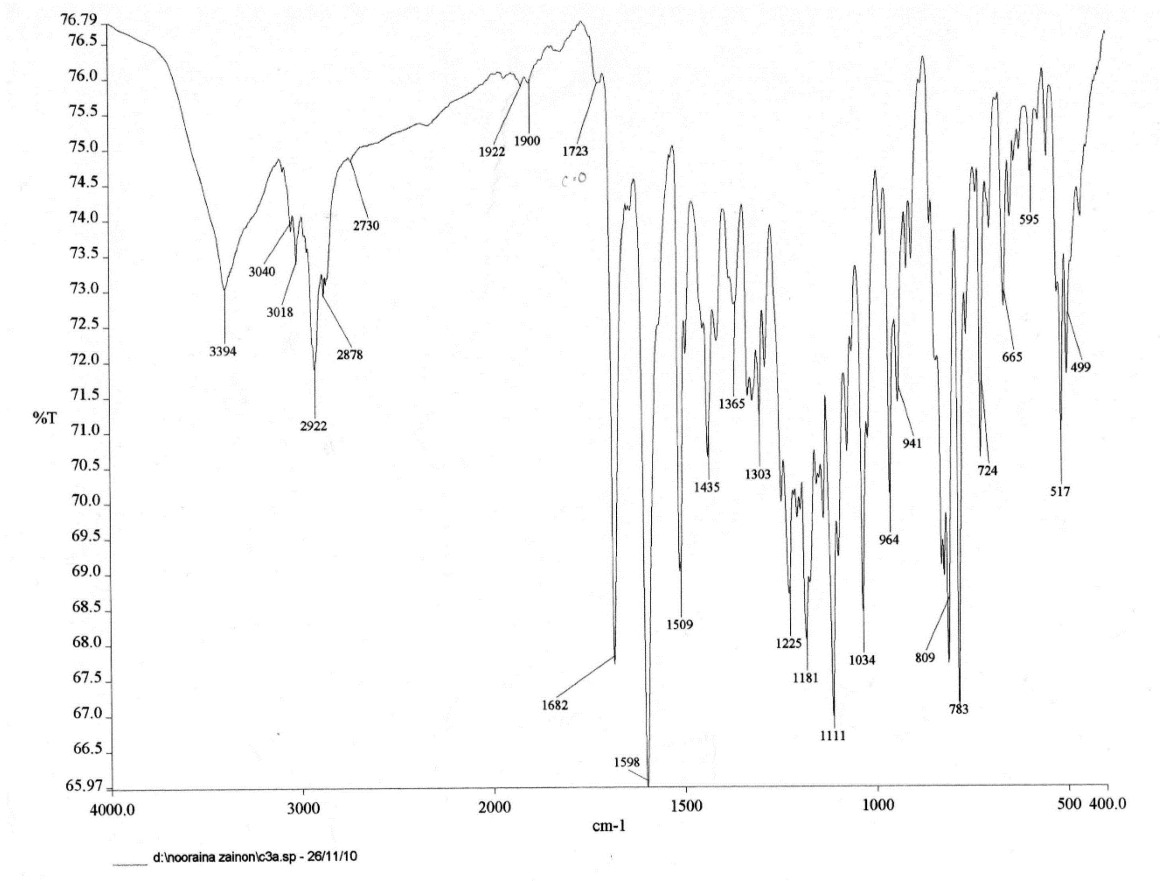

Figure 18. IR spectrum of **6i**.
